# A pH‐Switchable Cerium Single‐Atom Enzyme for Peri‐Implantitis Therapy via JAK‐STAT Signaling Axis‐Mediated Macrophage Reprogramming

**DOI:** 10.1002/advs.77002

**Published:** 2026-08-12

**Authors:** Xiaomin Xia, Haojie Wu, Xingyun Li, Lining Liu, Kaiqing Song, Jinpei Cui, Jie Liu, Jiaxuan Yin, Emtiaz Ahmed, Yusuke Yamauchi, Xue Li

**Affiliations:** ^1^ Department of Stomatology The Affiliated Hospital of Qingdao University Qingdao University Qingdao China; ^2^ School of Stomatology Qingdao University Qingdao China; ^3^ Institute of Materials for Energy and Environment College of Materials Science and Engineering Qingdao University Qingdao China; ^4^ Australian Institute for Bioengineering and Nanotechnology (AIBN) The University of Queensland Brisbane Queensland Australia; ^5^ Department of Materials Process Engineering Graduate School of Engineering Nagoya University Nagoya Japan

**Keywords:** Ce single‐atom enzyme, enzymatic relay, immunotherapy, peri‐implantitis, pH‐switchable catalysis

## Abstract

The development of high‐performance, environmentally adaptive enzyme‐mimicking catalysts for the treatment of peri‐implantitis (PI) is of great importance yet remains highly challenging. Herein, we report a high‐performance cerium single atom on B, N co‐doped carbon nanotubes enzyme (SAzyme), denoted as Ce‐BNC. Benefitting from its unique N/O‐coordinated electronic environment and reversible Ce^3+^/Ce^4+^ redox cycle, Ce‐BNC exhibits an intrinsic pH‐switchable catalytic function, with acidic conditions favoring peroxidase (POD)‐likeactivity for hydrogen peroxide (H_2_O_2_) activation and reactive oxygen species (ROS)‐mediated antibacterial effects, whereas relatively neutral conditions promote superoxide dismutase (SOD)‐ and catalase (CAT)‐like enzymatic cascades for ROS conversion and oxygen generation. Through this pH‐switchable catalytic transition, Ce‐BNC enables dynamic switching between oxidative antimicrobial activity and antioxidative microenvironment modulation. Mechanistically, this pH‐switchable enzymatic relay is thermodynamically governed by reduced Gibbs free‐energy barriers. This antioxidant effect further disrupts the M1 macrophage/IL‐6‐JAK/STAT signaling axis, thereby remodeling the inflammatory microenvironment. This work presents a microenvironment‐adaptive catalytic platform with programmable bimodal activities, offering a promising paradigm for the intelligent and staged treatment of inflammatory diseases.

## Graphical abstract

A pH‐switchable cerium single‐atom enzyme is developed for stage‐specific peri‐implantitis therapy. Acidic conditions trigger antibacterial peroxidase‐like activity, whereas neutral conditions activate an antioxidant superoxide dismutase ‐ and catalase ‐like enzymatic relay. This microenvironment‐adaptive catalytic strategy suppresses the IL‐6/JAK‐STAT signaling axis, remodels macrophage responses, and promotes inflammation resolution and tissue repair.

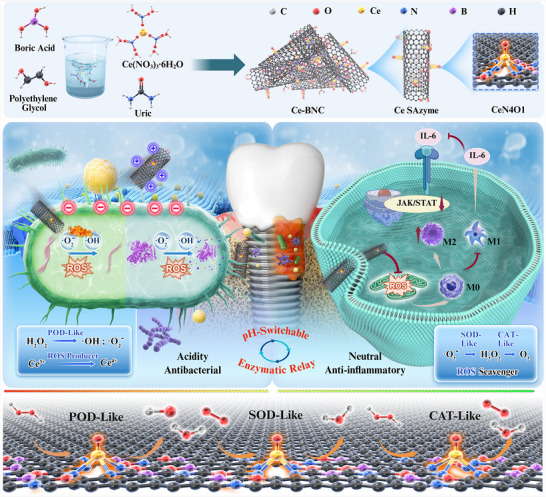

## 1 Introduction

Dental implants represent the gold standard for restoring the structural and functional integrity of missing dentition [1, 2]. Despite their high survival rates, the prevalence of peri‐implantitis (PI) remains alarmingly high, affecting approximately 20% of cases with a rising trajectory [3]. PI is characterized as a biofilm‐associated pathological condition involving inflammation of the peri‐implant mucosa and the progressive resorption of supporting bone [4]. Current clinical interventions, predominantly relying on mechanical debridement and local antibiotic administration, yield suboptimal outcomes due to the complexity of implant surfaces and the escalating threat of antimicrobial resistance [5, 6].

Local pH is an important feature of the bacterial infection and inflammatory microenvironment. Oral pathogenic biofilms can acidify their surroundings through carbohydrate fermentation and the accumulation of organic acids. In cariogenic biofilms, the local pH may decrease from a physiological average of approximately 6.7 to 4.5–5.5 [7], although this range should not be directly extrapolated to peri‐implant lesions, where the actual pH varies with microbial composition, disease stage, oxygen availability, and local buffering capacity [8]. Notably, in a multispecies peri‐implant biofilm model, the pH decreased from 6.97 in the planktonic bacterial suspension to 5.88 in the mature biofilm, supporting the presence of a mildly acidic biofilm microenvironment [9]. In parallel, inflamed peri‐implant tissues are characterized by local hypoxia, excessive reactive oxygen species (ROS) accumulation, inflammatory cell infiltration, and a reduction in pH. Such local acidification may influence bacterial metabolism, host inflammatory responses, and the catalytic or responsive behavior of microenvironment‐sensitive therapeutic materials. The pathological progression of PI also exhibits distinct temporal heterogeneity: the early phase is dominated by bacterial colonization and biofilm formation, creating an acidic microenvironment driven by microbial metabolism and local hypoxia [10]. Subsequently, pathogen invasion triggers host immune activation accompanied by explosive ROS generation, while the surrounding peri‐implant tissues generally maintain a near‐neutral pH. This intrinsic physiological pH gradient provides a pivotal endogenous cue for engineering intelligent, stimuli‐responsive therapeutic platforms. Consequently, developing “smart responsive materials” [11] that integrate dual‐modal therapeutic strategies with both pathogenic drivers, infection and inflammation, is of paramount importance yet remains a formidable challenge.

Nanozymes, defined as catalytically active sites engineered to mimic the functions of natural enzymes, represent a burgeoning frontier in biomedical science [12, 13, 14]. Recent advancements have extensively showcased the versatility of nanozymes in treating a broad spectrum of pathological conditions. In oncology, metal oxide‐ and noble metal‐based nanozymes (e.g., Fe_3_O_4_ CeO_2_, and Pt) have been strategically employed as catalase (CAT) mimetics. These agents alleviate local hypoxia by decomposing endogenous hydrogen peroxide (H_2_O_2_) into oxygen, thereby potentiating the efficacy of radiotherapy and photodynamic therapy [15, 16, 17]. Parallel to these pro‐oxidative applications, ROS‐scavenging nanozymes have emerged as potent tools for mitigating bacterial infections and chronic inflammation. For instance, CeO_2_ nanoparticles function as synergistic superoxide dismutase (SOD) and CAT mimics, effectively neutralizing excessive ROS. Such antioxidant interventions have demonstrated therapeutic success in models of sepsis, acute kidney injury, and inflammatory bowel disease by suppressing oxidative stress and modulating inflammatory cascades [18, 19]. Nanozyme‐mediated catalytic medicine, such as Fe_3_O_4_ (peroxidase‐like, POD‐like) and CeO_2_ (SOD‐like), has emerged as a promising paradigm for PI management, offering the potential to simultaneously address its dual pathogenic drivers: persistent microbial biofilms and dysregulated inflammation [18, 20]. Recent advances in nanomaterial‐based antibacterial applications have led to the emergence of numerous innovative therapeutic strategies [21, 22], among which enzyme‐mimicking catalytic therapies, particularly POD‐like activity‐induced ROS‐mediated antibacterial systems, have attracted considerable attention owing to their potent bactericidal efficacy and broad‐spectrum antimicrobial capabilities. However, a critical bottleneck resides in the lack of catalytic specificity [23, 24]. Conventional nanozymes often exhibit “promiscuous” enzymatic activities, where POD‐like and CAT‐like pathways coexist. In the intricate PI microenvironment, these simultaneous reactions may antagonize or compete for the same substrates, leading to “off‐target” effects or the generation of unintended byproducts, thereby failing to execute the required therapeutic sequence on demand [25].

Single‐atom enzymes (SAzymes), characterized by isolated metal atoms coordinated within functional support matrices, represent a significant advancement in nanozyme technology. Their defining feature is exceptional catalytic selectivity, achieved through well‐defined, atomically precise coordination environments that closely mimic the active sites of natural metalloenzymes [26, 27]. This structural precision, coupled with maximized atomic utilization, bridges the gap between homogeneous and heterogeneous catalysis, enabling both high reactivity and remarkable substrate specificity. The rational, bottom‐up design of SAzymes, guided by clear structure‐activity relationships, allows for tailoring their selective catalytic functions to diverse biomedical challenges [28, 29]. For instance, in oncology, SAzymes can be engineered with dominant POD‐like activity to selectively amplify chemodynamic therapy (CDT) within tumors. Their catalytic selectivity is key: they efficiently catalyze toxic radical generation in the tumor microenvironment while their secondary CAT‐like activity alleviates hypoxia, enabling synergistic and targeted combination therapies [30, 31]. By pushing catalytic activity to its theoretical limit, SAzymes harmonize the high reactivity and selectivity of homogeneous catalysts while inheriting the robust stability of heterogeneous catalysts [32, 33]. This positions them at the forefront of next‐generation artificial enzymes, paving the way for precision, multimodal therapies rooted in efficient and selective biocatalysis.

The distinct microenvironmental alterations are derived from the pathological progression of PI, which calls for the development of pH‐switchable catalytic cascade systems with the function of environment‐adaptive “enzymatic relay”. This on‐demand catalytic switching enables precise, sequential therapy from “attack” to “repair”. The unexplored potential of Ce single atoms lies in their high catalytic efficiency and superior redox switch sensitivity, which stems from a tailorable coordination environment. This unique feature, surpassing the capabilities of traditional nanoparticles, provides an efficient and precise mechanism for switching between antimicrobial and anti‐inflammatory states, a transition that has yet to be fully realized. In addition, further examining and exploring the immune mechanism of PI, identifying appropriate intervention targets and developing selective SAzyme to restore immune homeostasis, holds significant scientific and clinical value for the treatment of PI.

**SCHEME 1:**
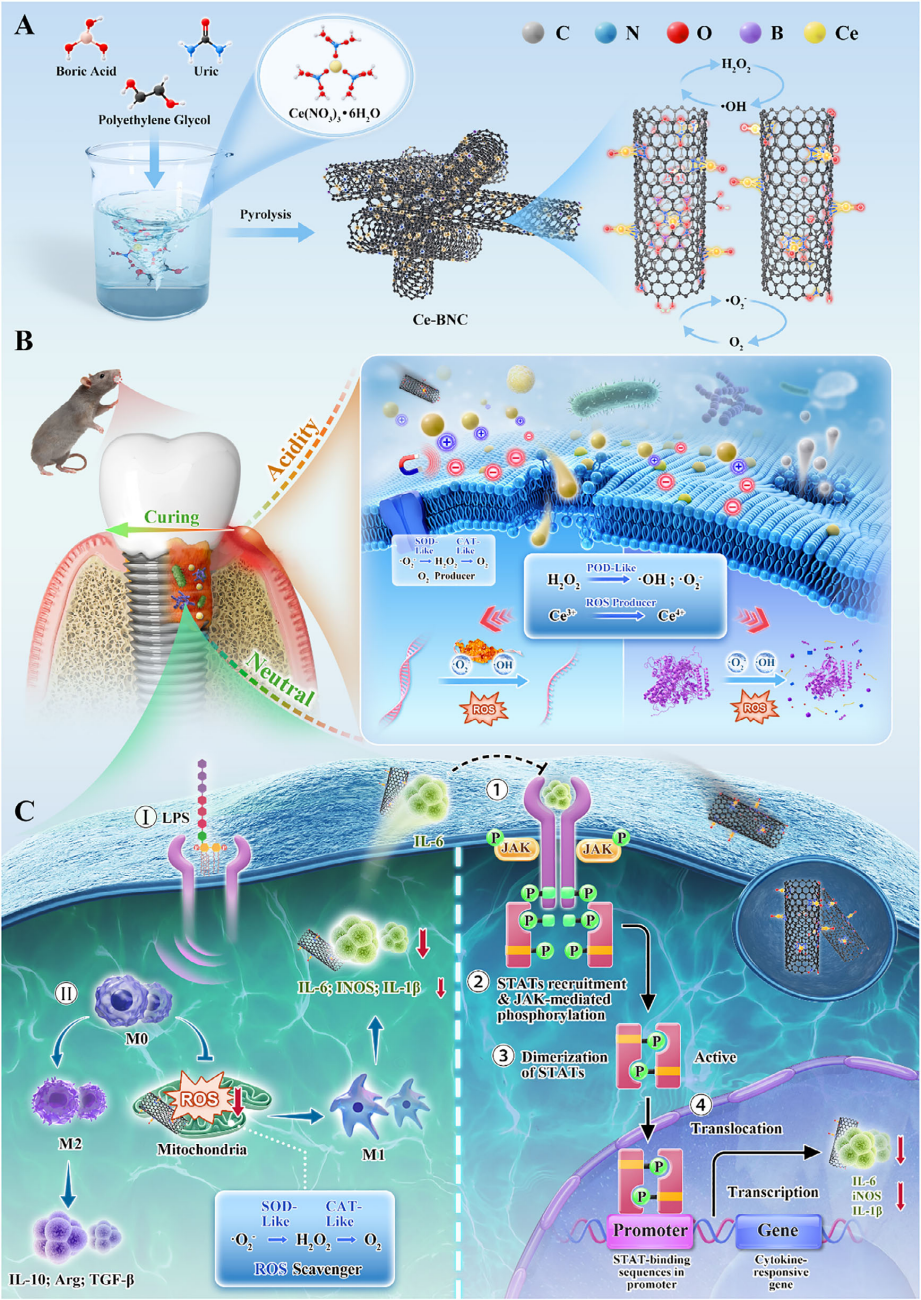
Schematic illustration of Ce‐BNC synthesis and of its proposed mechanisms for pathogen clearance and inflammation attenuation in peri‐implantitis.

Herein, a cerium‐based single‐atom enzyme (Ce‐BNC) was successfully engineered (Scheme 1). The unique pH‐switchable enzymatic relay mechanism of Ce SAzyme to orchestrate stage‐specific therapeutic interventions against PI was discovered and comprehensively studied by systematic physicochemical characterizations, density functional theory (DFT) calculations, and in vivo experiments. Under acidic conditions, Ce‐BNC exhibits dominant POD‐like activity that generates bactericidal ROS. Under near‐neutral conditions, its dominant catalytic activity shifts toward a SOD–CAT‐like relay, enabling the scavenging of excess ROS and the alleviation of oxidative stress. These pH‐dependent catalytic behaviors were demonstrated in vitro and supported by DFT calculations. The antioxidant efficacy disrupts the “M1 macrophage/IL‐6‐JAK/STAT” signaling axis and restores homeostasis by remodeling the immune microenvironment via precise inhibition of JAK‐STAT signaling. This work presents a high‐performance catalytic platform for PI management and exemplifies a SAzyme relay‐driven paradigm for designing intelligent SAzyme for complex inflammatory diseases.

## 2 Results and Discussion

### 2.1 Synthesis and Structural Characteristics of the Ce‐BNC SAzyme

**FIGURE 1:**
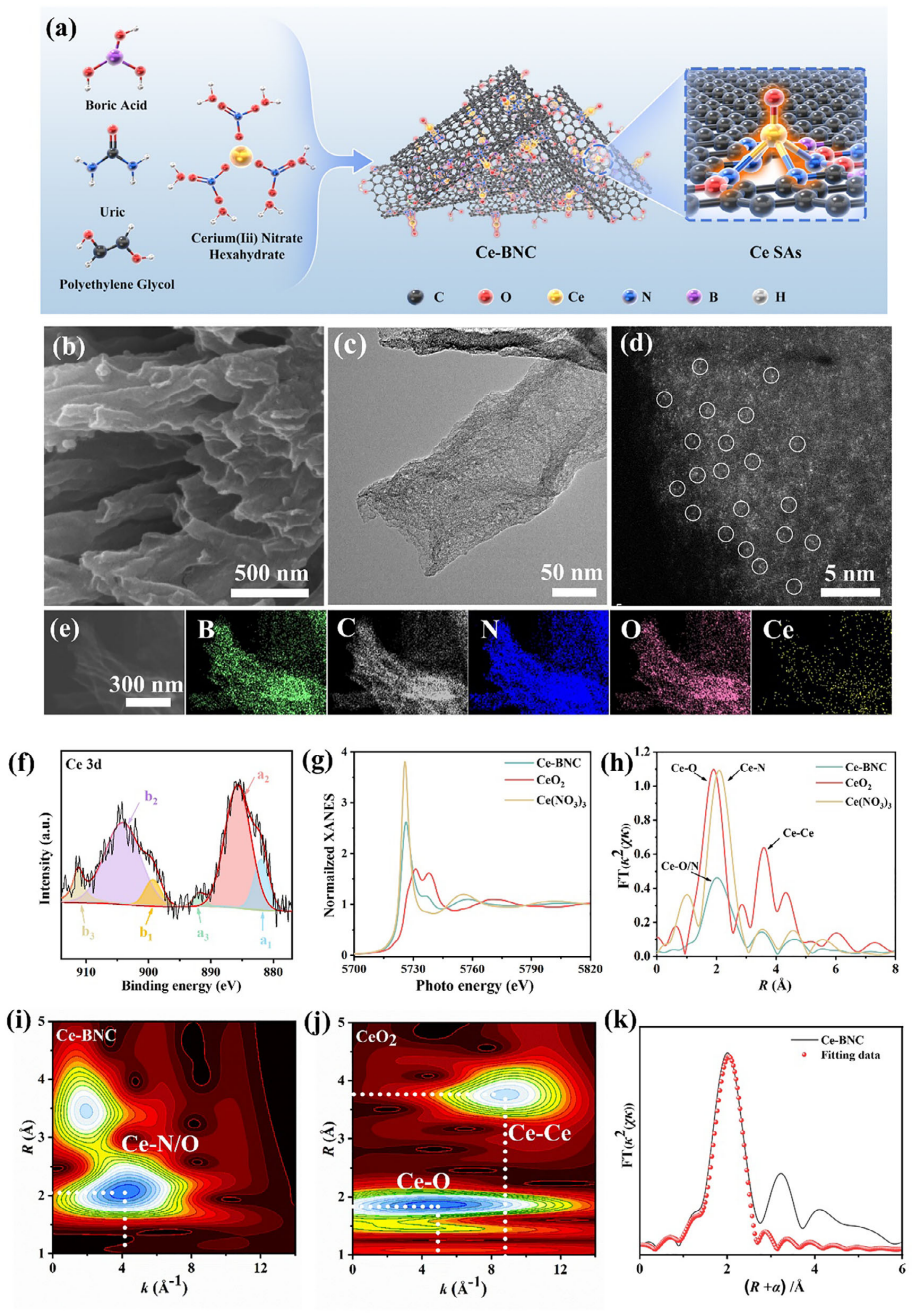
Characterizations of Ce‐BNC SAzyme. (a) Schematic demonstration of Ce‐BNC synthesis, (b) SEM image, (c) TEM image, (d) Aberration‐corrected HAADF‐STEM image, (e) STEM‐EDS elemental mapping, (f) deconvolution of Ce XPS spectra of Ce‐BNC SAzyme. (g) Normalized Ce *L*_3_‐edge XANES spectra of Ce‐BNC compared with reference samples. (h) Fourier‐transformed (FT) magnitude of the Ce *L*_3_‐edge EXAFS spectra in R‐space for Ce‐BNC and references. Wavelet transform (WT) of (i) Ce‐BNC and (j) CeO_2_. (k) First‐shell EXAFS fitting of Ce‐BNC in R‐space.

As schematically illustrated in Figure 1a, the Ce‐BNC SAzyme was synthesized via a facile solvothermal approach. Scanning electron microscopy (SEM) and transmission electron microscopy (TEM) characterizations (Figure 1b,c) reveal that Ce‐BNC retains a nanotube morphology, which is identical to that of the BNC material (Figure S1). The N_2_ physical sorption characterization shows that both BNC and Ce‐BNC retain abundant porous structures (Figures S2 and S3). Ce‐BNC exhibits a high specific surface area of 545.92 m^2^ g^−1^ and a total pore volume of 1.08 cm^3^ g^−1^, comparable to those of BNC, indicating that Ce incorporation did not substantially disrupt the porous carbon framework (Table S1). This porous hollow architecture is strategically advantageous, as it facilitates exposure of active sites and promotes mass transfer, thereby improving the catalytic efficacy [34]. The selected‐area electron diffraction (SAED) pattern shown in the inset of Figure S4 presents no distinct diffraction spots or sharp rings, indicating that Ce‐BNC has a poor crystalline structure. This observation is consistent with the broad X‐ray diffraction (XRD) peaks centered at approximately 24° and 43° in Figure S5, which is in accordance with the low‐graphitized carbon material [35]. Moreover, no additional diffraction peaks attributable to crystalline Ce‐containing phases were observed, indicating the absence of detectable crystallized Ce‐based nanoparticles. Consistent with these findings, the Fourier transform infrared (FTIR) spectra of Ce‐BNC show vibrational modes similar to those of the BNC substrate, with no detectable signal characteristic of crystalline CeO_2_ (Figure S6). To confirm the atomic dispersion of the Ce species, aberration‐corrected high‐angle annular dark‐field scanning transmission electron microscopy (HAADF‐STEM) was employed. As highlighted by the white circles in Figure 1d and Figure S7, high‐density bright spots are uniformly distributed across the support matrix, providing direct visual evidence of isolated Ce single atoms. Inductively coupled plasma optical emission spectroscopy (ICP‐OES) quantified the Ce loading to be 4 wt.% (Table S2). Energy‐dispersive spectroscopy (EDS) elemental mapping verified the homogeneous distribution of B, C, N, O and Ce within the nanotube framework (Figure 1e), underscoring the high dispersion of Ce element. These results collectively demonstrate the successful fabrication of a Ce single‐atom enzyme with a high atomic density and uniform dispersion.

To unveil the surface oxidation states and the electronic valence configurations of the Ce sites in Ce‐BNC, x‐ray photoelectron spectroscopy (XPS) measurements were carried out. As shown in Figure 1f, the Ce 3d XPS spectrum was deconvoluted into six distinct peaks. The peaks located at 904.13 eV (b_2_) and 885.75 eV (a_2_) are characteristic of Ce^3+^, while the remaining four peaks (a_1_, a_3_, b_1_ and b_3_) are assigned to Ce^4+^ [36]. Quantitative analysis reveals that cerium primarily exists in the +3 oxidation state, with a Ce^3+^/Ce^4+^ ratio of 3.3 (Table S3). Compared to the fully occupied 5s^2^5p^6^ configuration of Ce^4+^, the 5s^2^5p^6^4f^1^ configuration of Ce^3+^ is expected to facilitate electron exchange and proton coupling, thereby enhancing the adsorption of reactant molecules [37]. The precise electronic structure and local coordination of Ce were further scrutinized using x‐ray absorption spectroscopy (XAS). In the Ce *L_3_*‐edge x‐ray absorption near‐edge structure (XANES) spectra (Figure 1g), Ce‐BNC displays a prominent single white line peak at 5726.9 eV. This feature closely resembles the Ce (NO_3_)_3_ standard but is markedly distinct from the doublet‐peak profile of CeO_2_, corroborating that the valence state of Ce in Ce‐BNC is predominantly +3. The *k* ^3^‐weight EXAFS spectrum in R‐space of Ce‐BNC (Figure 1h) exhibits a primary coordination peak at 2.12 Å, attributed to the Ce‐N/O first shell. Notably, the absence of a characteristic Ce–Ce scattering peak at 3.68 Å confirms the atomic dispersion of Ce, consistent with our HAADF‐STEM observations [36, 38]. The wavelet transforms (WT‐EXAFS) plots (Figure 1i,j) further substantiate the absence of Ce–Ce coordination, while the intensity for Ce‐BNC lies at 4.2 Å^−1^, which is significantly shifted compared to the Ce─O bond in CeO_2_. This validates the single‐atomic state of Ce with a more sophisticated coordination structure. Quantitative FT‐EXAFS fitting was performed to elucidate the local coordination environment of Ce in Ce‐BNC (Figure 1k), and the corresponding fitting parameters are summarized in Table S4. The results indicate that the Ce centers are coordinated with both N and O atoms in the carbon matrix. Specifically, the fitted coordination numbers were 3.90 for the Ce–N path at 2.61 ± 0.03 Å and 1.37 for the Ce–O path at 2.46 ± 0.17 Å, suggesting an approximately N_4_O_1_ coordinated local environment around the Ce center. The low *R*‐factor of 0.0117 indicates good agreement between the experimental and fitted spectra. Combined with the absence of an evident Ce–Ce coordination contribution and the complementary microscopic and spectroscopic results, these findings support the atomic dispersion of Ce species and a CeN_4_O_1_‐like local coordination structure.

To consolidate the first shell coordination structure, DFT calculations were further applied to simulate the possible Ce‐coordinated models, including CeO_5_, CeO_4_N_1_, CeO_3_N_2_, CeO_2_N_3_, CeO_1_B_4_, CeO_1_B_3_N_1_, CeO_1_B_2_N_2_, CeO_1_B_1_N_3_, and CeN_4_O_1_ to compare their formation energies. As shown in Figure S8, among all the considered configurations, the CeN_4_O_1_ model exhibited the lowest formation energy of 0.37 eV, whereas B‐containing first‐shell models were energetically not favorable. Therefore, the local structure of Ce in Ce‐BNC is assigned as a CeN_4_O_1_‐like configuration with B atoms located at the long‐range shells. At the same time, B1s XPS analysis (Figure S9) shows the deconvoluted XPS peaks of B–C, B–N and B–O, further indicating that B is incorporated into the carbon framework without direct coordination with Ce centers. By the charge density analysis (Figure S10) of the CeN_4_O_1_ entity, it could be seen that Ce sites are electronically enriched with electrons donating from the ligating atoms, however, the electronic communication is significantly attenuated between Ce with the long‐range shell atoms. Therefore, the CeN_4_O_1_ structure was applied as the model in the following analysis and the mechanistic study. The optimized three‐dimensional (3D) geometry of the CeN_4_O_1_ active site is presented in Figure S11, providing both top and side views to demonstrate the local planarity and the spatial arrangement of the isolated Ce center within the carbonaceous framework.

### 2.2 The Enzymatic Mimetic Catalytic Performance

The high biological efficacy of SAzymes relies on their versatile enzyme‐mimicking activities. SAzymes with the highest antibacterial activity typically exhibit POD‐like activity. However, CAT‐like activity and SOD‐like activity are also important, which are responsible for the inflammation alleviation [39, 40]. Consequently, the multi‐functional enzymatic profiles of the Ce‐BNC SAzyme in vitro were systematically evaluated.

**FIGURE 2:**
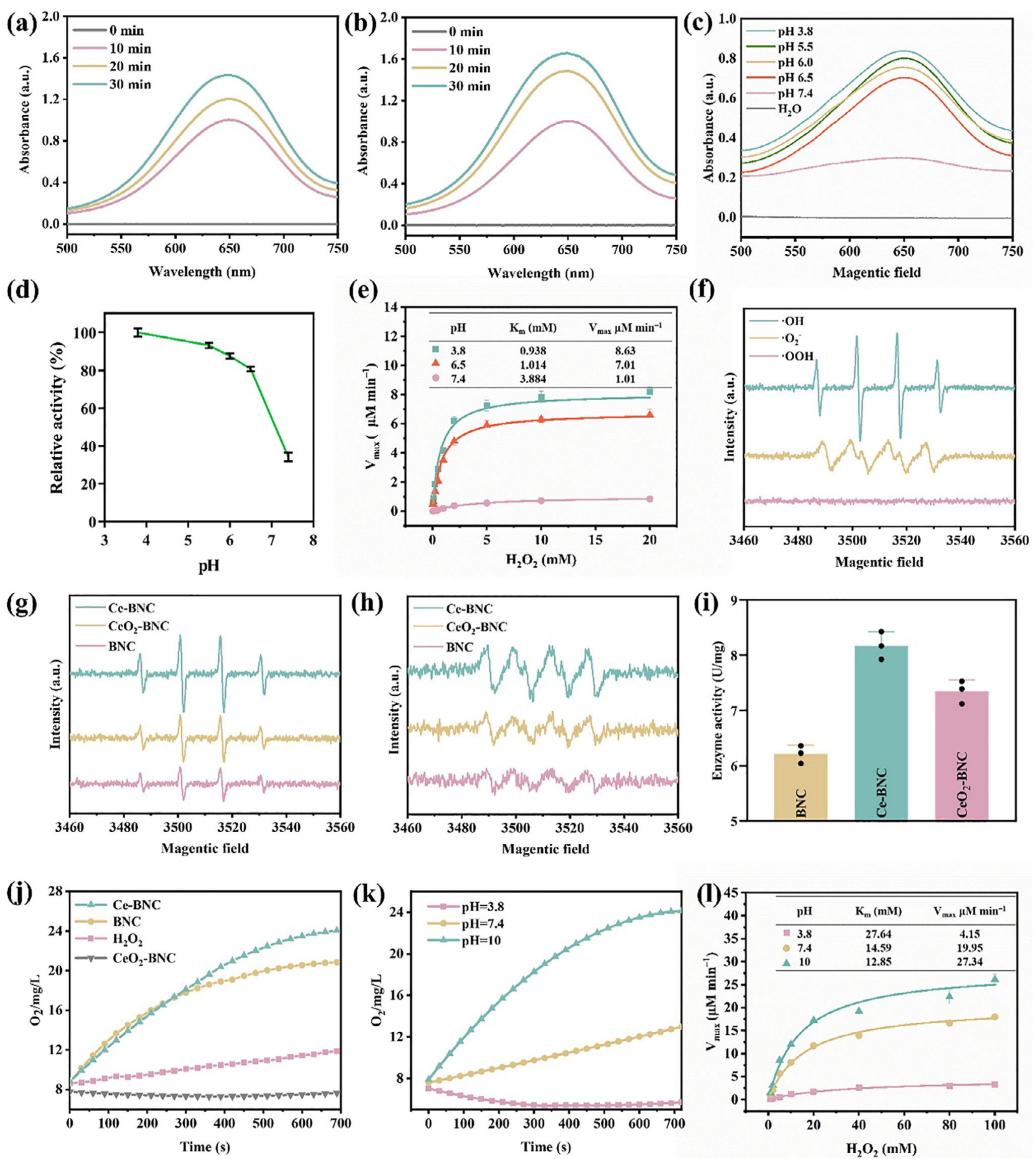
The enzymatic mimetic catalytic activity evaluation. Time‐dependent UV–vis absorption spectra of ox‐TMB catalyzed by (a) BNC and (b) Ce‐BNC under acidic conditions. (c) pH‐dependent UV–vis absorption changes of ox‐TMB in the presence of Ce‐BNC. (d) Relative POD‐like activity of Ce‐BNC at different pH values. The POD‐like catalytic activity was normalized to the maximum activity observed under the optimal pH condition. (e) Michaelis−Menten kinetic analysis for Ce‐BNC with H_2_O_2_ as a substrate for POD‐like activity. The ·OH, ·O_2_^−^ and ·OOH generated during the activation of H_2_O_2_ by (f) Ce‐BNC. ESR spectra of the DMPO‐trapped spin adduct for (g) ·OH and (h) ·O_2_^−^ produced by BNC, CeO_2_‐BNC and Ce‐BNC. (i) SOD‐like activities of BNC, Ce‐BNC and CeO_2_‐BNC. (j) Time dependent oxygen generation in CAT‐like for H_2_O_2_, BNC, Ce‐BNC and CeO_2_‐BNC. (k) pH‐dependent oxygen evolution kinetics of Ce‐BNC. (l) Michaelis−Menten kinetic analysis for Ce‐BNC with H_2_O_2_ as a substrate for CAT‐like activity. Data is presented as the mean ± SD from three independent experiments (*n* = 3).

To verify the POD‐like activity of Ce‐BNC, the H_2_O_2_‐mediated oxidation of TMB was monitored. The generation of ·OH radicals led to the formation of blue‐colored ox‐TMB, whose characteristic absorption at 652 nm was recorded using UV–vis spectrophotometry [41]. Factors affecting enzyme activity, such as time, pH value and the amount of Ce dopant were further evaluated. Ce‐BNC (Figure 2b) exhibits a remarkable time‐dependent POD‐like catalytic reactivity, superior to that of BNC (Figure 2a), which may be due to the activity contribution of the Ce active sites. In alignment with established models for single‐atom POD mimics, Ce‐BNC exhibits pH‐switchable catalytic performance [42]. With the increase of pH value, POD‐like activity gradually decreases, and it almost reaches near‐baseline levels under neutral conditions (Figure 2c). Ce‐BNC exhibits optimal POD‐like properties at a pH of 3.8, while Ce‐BNC retained approximately 96%, 91%, and 84% of its maximal activity at pH 5.5, 6.0, and 6.5, respectively (Figure 2d). To quantitatively evaluate the POD‐like catalytic kinetics of the material at different pH values, the initial reaction rates were measured using H_2_O_2_ as the substrate and fitted to the Michaelis–Menten model (Figure 2e). The material exhibits the highest catalytic activity at pH 3.8, with a *K_m_* of 0.938 mm and a *V_max_* of 8.63 µm min^−1^. At pH 6.5, catalytic activity remains relatively high, with a *K_m_* of 1.014 mm and a *V_max_* of 7.01 µm min^−1^, whereas it is markedly reduced at pH 7.4. These results demonstrate that the material exhibits enhanced POD‐like activity under acidic conditions. As the amount of Ce increases, the increase in POD‐like activity shows the volcano‐type trend (first increasing and then decreasing), with 4 wt.% Ce content delivering the best performance and the representative traditional nanoparticles CeO_2_‐BNC exhibits the lowest enzymatic activity (Figure S12a). This is attributed to the fact that increasing Ce content introduces more cerium precursor, leading to the formation of CeO_x_ clusters [43]. Electron spin resonance (ESR) measurements with 5, 5‐dimethyl‐1‐pyrroline N‐oxide (DMPO) further confirmed the POD‐like activity of Ce‐BNC. Specifically, Figure 2f demonstrates its ability to catalyze H_2_O_2_ decomposition into hydroxyl radical (·OH) and superoxide radical (·O_2_^−^) at pH 3.8. Comparative analyses reveal that Ce‐BNC outperforms both pristine BNC and the CeO_2_‐BNC nanoparticle control (Figure 2g,h). In addition, both BNC and Ce‐BNC can oxidize TMB to blue oxidized TMB (ox‐TMB) (Figure S12b).

Subsequently, the antioxidant capacity of the Ce‐BNC SAzyme was quantified using the WST‐8 colorimetric assay. In distinct contrast to the acidic POD‐like activity, Ce‐BNC exhibits strong SOD‐like activity than BNC and CeO_2_‐BNC under near‐neutral conditions (Figure 2i). Intriguingly, the SOD‐like activity shows a decreasing tendency with the increase of Ce loading (Figure S12c). As was reported, the SOD‐like performance of cerium‐based materials is intrinsically linked to the reversible transition between Ce^3+^ and Ce^4+^ [44]. Specifically, the scavenging of ·O_2_^−^ is typically mediated by the oxidation of Ce^3+^ to Ce^4+^. However, during the high‐concentration loading process, Ce at a higher content (e.g., CeO_2_‐BNC) may preferentially exist in the more stable Ce^4+^ state, due to variations in surface energy or the influence of the preparation environment [45], which provides an explanation for the above experimental findings. Although SOD‐like activity is essential for protecting biological systems from ·O_2_^−^ mediated oxidative damage, the resulting enzymatic byproduct—H_2_O_2 –_ remains a potential threat to genomic stability and lipid integrity if it is not further neutralized [46]. This underscores the necessity of the CAT‐like activity in our SAzyme system to complete the antioxidant enzymatic relay.

The subsequent detoxification of H_2_O_2_ within biological systems is primarily mediated by CAT‐like activity, which catalyzes the disproportionation of H_2_O_2_ into H_2_O and O_2_. By quantifying the dissolved O_2_ production rates, both BNC and Ce‐BNC exhibit robust efficacy in decomposing H_2_O_2_ to generate O_2_. This may be due to the competition among multiple substrates, resulting in complex enzymatic activity results for the CeO_2_‐BNC (Figure 2j). Among the variants with different Ce loadings and support matrices, the 1% Ce‐BNC (4 wt.% Ce content) demonstrates the highest CAT‐like potency (Figure S12d), displaying a trend consistent with POD‐like activity. When exploring the optimal pH value for the activity of the CAT‐like activity, it can be found that the SAzyme shows excellent performance under neutral and alkaline conditions (Figure 2k), which is consistent with the previous reports [46, 47]. Similarly, the CAT‐like catalytic kinetics of the Ce‐BNC at different pH values were evaluated by measuring the initial reaction rates using H_2_O_2_ as the substrate and fitting the data to the Michaelis–Menten model (Figure 2l). The material exhibits the highest catalytic activity at pH 10, with a *K_m_* of 12.85 mm and a *V_max_* of 27.34 µm min^−1^. At pH 7.4, the *K_m_* and *V_max_* values are 14.59 mm and 19.95 µm min^−1^, respectively, whereas the catalytic activity is markedly reduced at pH 3.8. These results indicate that the material exhibits enhanced catalytic activity under neutral and alkaline conditions.

To further evaluate the catalytic stability of Ce‐BNC, a repeated‐use experiment was performed using its POD‐like activity as a representative model, in which the catalyst was recovered after each reaction and reused for successive catalytic cycles. After five consecutive catalytic cycles, the characteristic signal intensity remained nearly unchanged compared with that of the first cycle, indicating that Ce‐BNC retains stable enzyme‐like activity (Figure S13). In addition, Ce‐BNC retains the nanotube structure and aberration‐corrected HAADF‐STEM characterization of the post‐ reacted sample shows that the isolated Ce atomic sites are still uniformly dispersed on the BNC matrix, without obvious aggregation or nanoparticle formation (Figure S14). These results confirm the good catalytic durability and structural stability of Ce‐BNC after repeated catalytic reactions.

The superior enzyme‐mimetic activity of Ce‐based materials originates from the synergy between their chemical redox cycle and electronic structure. Compared to the nearly fully occupied 5s^2^5p^6^ configuration of Ce^4+^ in traditional nanoparticle‐CeO_2_, the 5s^2^5p^6^4f^1^ configuration of Ce^3+^ in Ce‐BNC is expected to facilitate electron exchange and proton coupling, thereby enhancing the adsorption of reactant molecules such as H_2_O_2_, ·O_2_^−^, and H_2_O [37]. This specific electronic arrangement facilitates efficient electron exchange and proton coupling. Taking together, these results demonstrate that the Ce‐BNC SAzyme exhibits superior POD‐like catalytic activity as well as enhanced SOD‐ and CAT‐like antioxidant activities compared with both BNC and conventional CeO_2_ nanoparticles. Notably, a sophisticated catalytic bifurcation is established: the pro‐oxidative POD‐like activity is selectively triggered under acidic conditions (pathological PI state), whereas the antioxidative SOD‐CAT‐like activity is predominantly activated in neutral environments (inflammatory phase of PI). This pH‐switchable bimodal mechanism provides a robust biochemical foundation for the intelligent and microenvironment‐adaptive treatment of PI.

### 2.3 Enzymatic Relay Catalytic Mechanism of pH‐Switchable Ce‐BNC

The distinct enzyme‐like activities exhibited by Ce‐BNC under different catalytic conditions may be closely related to its compositional characteristics. According to the current literature, most studies on metal SAzymes have focused primarily on identifying the optimal pH for catalytic activity [48, 49, 50], whereas the underlying mechanisms responsible for activity switching under different pH conditions remain insufficiently understood. Therefore, in this study, we further investigated Ce‐BNC to elucidate the origin of its pH‐switchable enzymatic selectivity.

**FIGURE 3:**
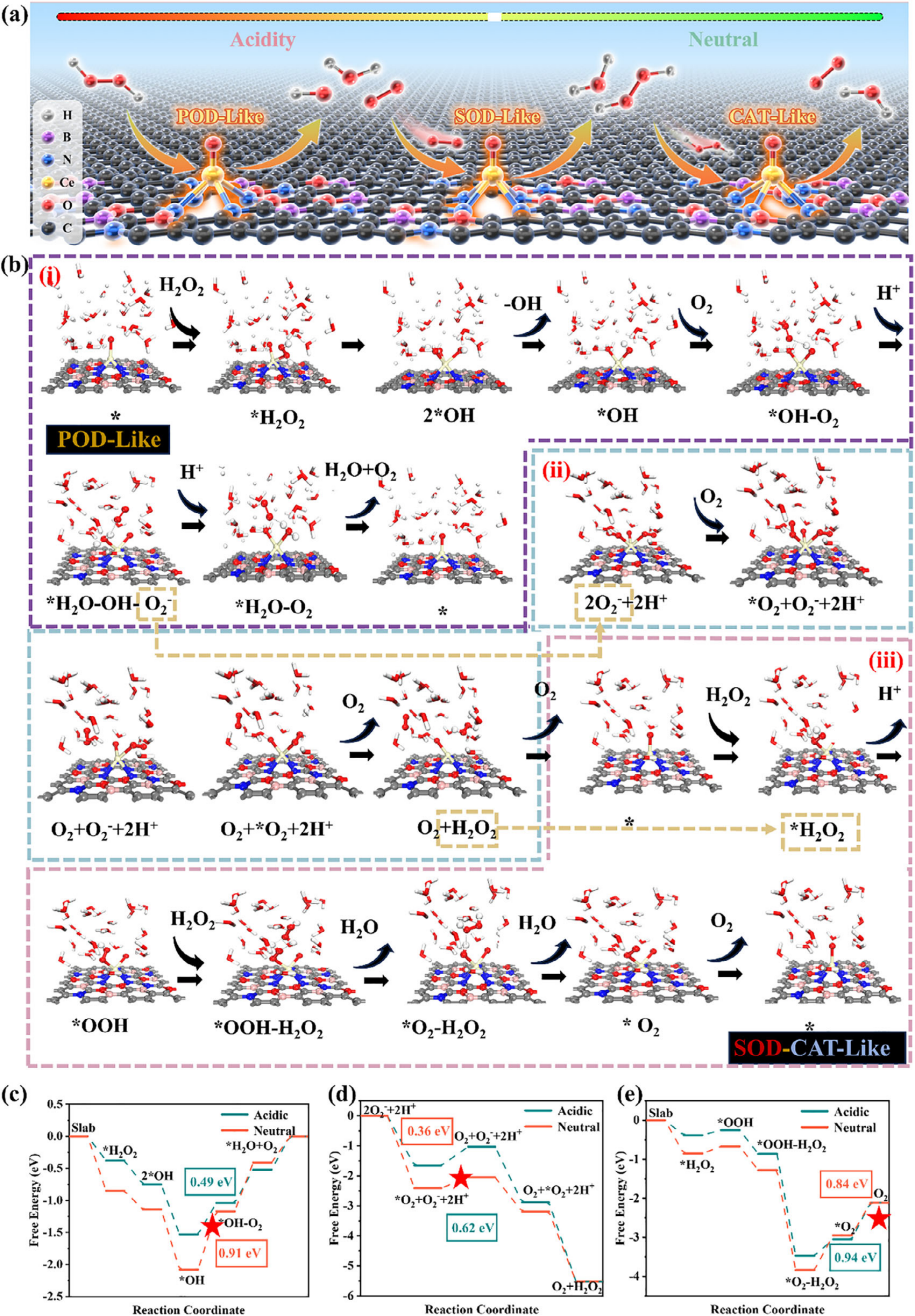
DFT calculations and atomistic insights into pH‐switchable catalytic selectivity. (a) pH‐switchable of the POD‐, SOD‐, and CAT‐like activity of Ce‐BNC. (b) Ce‐BNC acts as a simulated pathway for the SOD‐, POD‐, and CAT‐like activity. Calculated thermodynamic energy landscapes for the (c) POD‐, (d) SOD‐, and (e) CAT‐like catalytic cycles. Red five‐pointed star symbolizes the RDS.

Study employed DFT to meticulously map the potential energy surfaces (PES), focusing on the ground‐state configurations and transition‐state geometries of reaction intermediates under distinct pH conditions first. Figure 3a summarizes the pH‐switchable responses of Ce‐BNC's POD, SOD‐CAT‐like activities. The POD‐like activity pathway (Figure 3b(i)) shows that when the H_2_O_2_ molecule adsorbs onto the active Ce site in Ce‐BNC SAzyme, the O─O bond breaks. One of the ·OH subsequently dissociates from the catalyst surface to form an active ·OH, while the other ·OH remains adsorbed at the active site. Under acidic conditions, this adsorbed ·OH intermediate undergoes protonation to form an adsorbed ^*^H_2_O species [42]. Subsequent desorption of H_2_O regenerates the catalyst surface to its initial state. The rate‐determining step (RDS) of this cycle is identified as the coupling of the adsorbed ·OH, which facilitates the subsequent redox turnover. Remarkably, the Gibbs free energy barrier for this RDS under acidic conditions is merely 0.49 eV, which is approximately half of that required under neutral conditions (Figure 3c). This is attributed to two factors: first, the POD‐like activity process requires proton (H^+^) transfer, which can be more readily provided in acidic environments [51]. Second, the high Ce^3+^/Ce^4+^ ratio in Ce‐BNC enables Ce^3+^ to donate electrons that facilitate H_2_O_2_ decomposition. Therefore, POD‐like catalytic reactions are better triggered in acidic conditions.

Under neutral conditions, the excellent SOD‐CAT‐like activity for Ce‐BNC was motivated. As the reaction pathways shown in Figure 3b(ii, iii), CAT‐like activity manifests as the facilitated degradation of H_2_O_2_, a process potentially governed by a series of discrete chemical intermediates (Figure 3e). In terms of SOD‐like activity process, we hypothesize that the mechanism involving Ce single atoms is similar to the general catalytic scheme of the SOD‐like activity (Figure 3d) [52]. Significantly, the CAT‐like cycle concludes with the release of molecular oxygen. The process occurs concurrently and synergistically with SOD‐like activity, creating an interpenetrated catalytic network (Figure 3b(ii, iii)). Analysis of the potential‐determining step and intermediate configurations reveals a unique dynamic behavior: the rotation of ·O_2_^−^ or O_2_ molecules is accompanied by subtle positional adjustments of the coordinated Ce atoms. This structural flexibility allows the Ce centers to optimize their coordination geometry during the reaction, providing a quantum‐chemical rationale for the SAzyme's superior performance in SOD‐like disproportionation [53]. By facilitating this enzymatic relay, the Ce‐BNC SAzyme effectively transitions from a pro‐oxidant in acidic conditions to a potent antioxidant under neutral physiological conditions, thereby promoting the resolution of oxidative stress.

Figure 3e illustrates the optimized geometric structures of key reaction intermediates and the corresponding Gibbs free energy profiles for the CAT‐like decomposition of H_2_O_2_. In this pathway, an H_2_O_2_ molecule first adsorbs onto the isolated Ce site and undergoes deprotonation to form a stable ^*^OOH intermediate. Subsequently, a second H_2_O_2_ molecule abstracts a proton from ^*^OOH, yielding ^*^O_2_ and ^*^H_2_O species. The catalytic cycle concludes with the desorption of H_2_O and O_2_, thereby regenerating the CeN_4_O_1_ active site. O_2_ desorption was identified as the thermodynamically limiting step of the CAT‐like pathway. The corresponding uphill free‐energy change was lower under neutral conditions than under acidic conditions (0.84 vs 0.94 eV), indicating that O_2_ release is more favorable at neutral pH and supporting the enhanced CAT‐like activity of Ce‐BNC under physiological conditions. Similarly, the SOD‐like activity exhibits enhanced thermodynamic feasibility under neutral conditions (Figure 3d). While the H_2_O_2_ decomposition process remains inherently stable across a range of pH values, the SOD‐CAT‐like enzymatic relay is markedly more efficient in neutral environments. This is facilitated by the high stability and rapid Ce^3+^/Ce^4+^ redox cycling within the N_4_O_1_ coordination sphere, which maintains the integrity of the active centers.

Differential charge density and projected density of states (PDOS) analyses were performed for the optimized CeN_4_O_1_ active site. The differential charge density map shows obvious charge redistribution around the Ce center and the first‐shell N/O ligands, indicating strong electronic coupling within the CeN_4_O_1_ coordination motif (Figure S10). The N/O coordination atoms form a stable coordination structure with the single‐atom Ce center, significantly regulating its local electronic structure and interfacial charge behavior. On one hand, the N/O coordination enhances the polarization of the electronic cloud surrounding the Ce center, thereby modulating the reversible Ce^3+^/Ce^4+^ redox transition. On the other hand, this coordination environment optimizes the adsorption energetics of key reaction intermediates, enabling a balanced “moderate adsorption–reversible desorption” behavior that effectively prevents active‐site poisoning caused by overly strong binding. To further clarify the electronic origin of the pH‐switchable catalytic behavior, differential charge density and PDOS analyses were performed to explore the electronic interaction of Ce active site with the reaction intermediates. The PDOS results (Figure S15) further reveal that, for the ^*^OH intermediate, the acidic condition leads to stronger overlap between Ce states and the O states of the adsorbed intermediates near the Fermi level, suggesting enhanced Ce–O electronic interaction and more efficient electron transfer. This electronic coupling facilitates the activation of oxygen‐containing intermediates and promotes the Ce^3^^+^/Ce^4^^+^ redox cycle, thereby favoring H_2_O_2_ activation and ^*^OH generation. In contrast, for the ^*^O_2_ + O_2_ + 2H^+^ and ^*^O_2_ intermediates, the neutral condition shows a more moderate Ce–O orbital hybridization near the Fermi level, which can maintain electron transfer while avoiding excessive intermediate binding, thereby facilitating O_2_ generation.

In summary, the above results that the Ce‐BNC SAzyme possesses a microenvironment‐adaptive catalytic capability, selectively triggering POD‐like or SOD‐CAT‐like in response to the local pH variation. Those theoretical results provide valuable guidance for the subsequent in vitro and in vivo biomedical evaluations.

### 2.4 Biological Safety and Antioxidant Properties of Ce‐BNC

#### 2.4.1 Biocompatibility and Intracellular ROS Scavenging In Vitro

**FIGURE 4:**
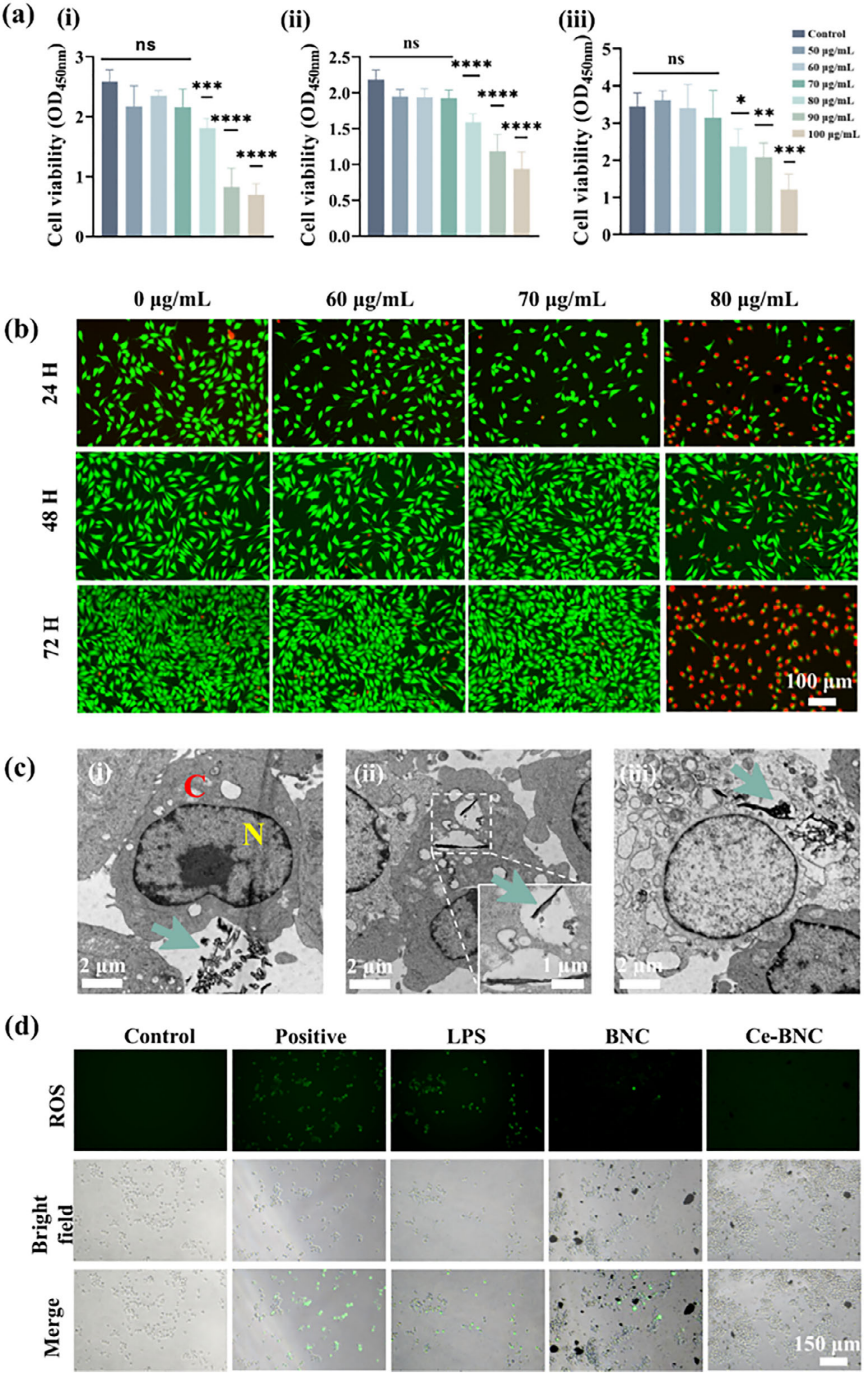
In vitro biocompatibility and antioxidant efficacy of Ce‐BNC. (a: i–iii) Cell viability of mouse fibroblast cell (L929) following incubation with Ce‐BNC at varying concentrations (0, 50, 60, 70, 80, 90, and 100 µg mL^−1^) for 24, 48 and 72 h, respectively. Cells without Ce‐BNC (0 µg mL^−1^) served as the blank control. Horizontal bars indicate statistical significance compared to the blank control. (*n* = 6, ^*^ *p* < 0.05, ^**^ *p* < 0.01, ^***^ *p* < 0.001, ^****^ *p* < 0.0001, ns: not significant). (b) Representative live/dead fluorescence staining images of L929 after treatment with Ce‐BNC. (c: i–iii) Bio‐TEM images of Ce‐BNC. Enlarged views (bottom) from the boxed regions show the internalization of SAzyme with blue arrows indicating the accumulation of Ce‐BNC within the cytoplasm (C) and near the nucleoplasm (N). (d) Evaluation of intracellular ROS levels via DCFH‐DA staining. All images within the same image set were acquired at the same magnification and that the scale bar shown in the final image applies to the entire set.

The biomedical application of multifunctional nanozymes is strictly contingent upon their biocompatibility and systemic safety. The study first performed dose‐dependent cytotoxicity assays to establish the safety profile of Ce‐BNC and BNC. As illustrated in Figure 4a,b and Figures S16 and S17, Ce‐BNC exhibited good cytocompatibility in L929 and RAW264.7 cells at concentrations up to 70 µg mL^−1^, while BNC remained non‐cytotoxic below 80 µg mL^−1^. Comparisons among different material concentrations at the same incubation time point. The CCK‐8 results show that cell viability remains at a high level across all tested concentrations, and no obvious cytotoxicity is induced by either Ce‐BNC or BNC. The live/dead staining further confirms that most cells maintain normal morphology and survival state, with only a negligible proportion of dead cells after incubation with the materials. Quantitative hemolysis assays further corroborate these findings: at Ce‐BNC concentrations of 70, 80, and 90 µg mL^−1^, the hemolysis rates are 4.02 ± 0.59%, 4.83 ± 0.28%, and 5.73 ± 0.23%, respectively (Figure S18). Adhering to the established safety threshold of < 5% hemolysis for blood‐contacting materials [54]. The concentration of 70 µg mL^−1^ was selected for all subsequent biological evaluations.

Effective cellular internalization is a fundamental requirement for nanozyme to exert their biological effects [55]. Bio‐TEM imaging of cells treated with Ce‐BNC for 24 h reveals distinct uptake patterns. As shown in Figure 4c, the SAzyme is localized within the cytoplasm, primarily sequestered inside endocytic vesicles. Characteristic membrane invagination and fusion events are observed, suggesting that the internalization of Ce‐BNC is mediated by endocytosis. At supra‐optimal concentrations, an excessive accumulation of intracellular vesicles is discovered, which may physically impede organelle function and trigger cytotoxic cascades as shown in Figure 4c(iii) [56].

Given the potential of high‐level ROS to induce irreversible oxidative damage to DNA and lipids [57], the intracellular ROS scavenging efficiency of Ce‐BNC was scrutinized using a lipopolysaccharide (LPS)‐induced inflammation model in RAW264.7 macrophages. The Positive group corresponds to the positive control provided by the commercial ROS detection kit, which was included to verify the validity and responsiveness of the staining system. Intracellular ROS levels were quantified using the 2',7'‐dichlorofluorescin diacetate (DCFH‐DA) fluorescent probe. As is depicted in Figure 4d, the LPS‐stimulated positive control exhibits intense green fluorescence, signaling robust ROS generation. In contrast, treatment with Ce‐BNC leads to a dramatic attenuation of the fluorescent signal, with the green intensity being virtually undetectable in the Ce‐BNC group. ROS levels in LPS‐stimulated RAW264.7 macrophages were analyzed quantitatively by flow cytometry using DCFH‐DA (Figure S19). The proportion of ROS‐positive cells increased markedly from 2.56% in the control group to 27.80% after LPS stimulation, confirming the induction of oxidative stress. BNC treatment moderately reduced the ROS‐positive population to 21.00%, whereas Ce‐BNC treatment further decreased it to 7.79%. These results demonstrate that Ce‐BNC effectively suppresses LPS‐induced intracellular ROS accumulation in macrophages and exhibits substantially stronger antioxidant activity than BNC alone. These results collectively demonstrate that Ce‐BNC not only possesses superior biocompatibility but also functions as a potent antioxidant cyto‐protector by leveraging its synergistic SOD‐CAT‐like enzymatic relay activities to restore intracellular redox homeostasis.

#### 2.4.2 Microenvironment Biocompatibility In Vivo and Histopathological Evaluation

The metabolic fate and degradability of nanoparticles within the biological milieu are critical determinants of their long‐term biosafety [58]. To evaluate the in vivo tissue biocompatibility of Ce‐BNC, mouse skin infection model was constructed, with the SAzyme administered via subcutaneous implantation around the wound periphery (Figure S23a). On the 10th day post‐surgery, pathological examinations were harvested on the wound tissue.

**FIGURE 5:**
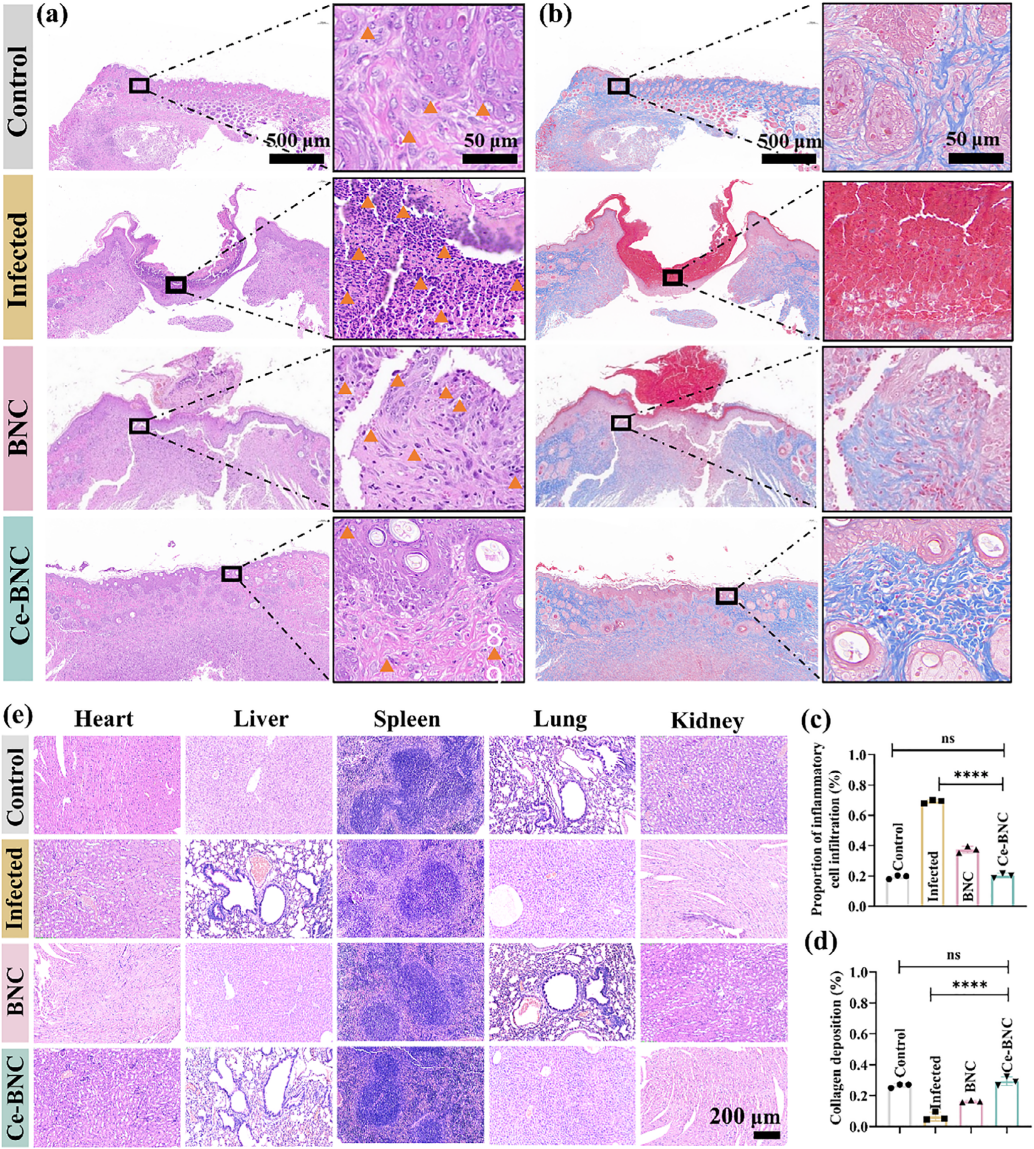
In vivo biocompatibility and histopathological evaluation of Ce‐BNC. (a) H&E and (b) Masson's trichrome staining of local peri‐lesional tissue at day 10 post‐intervention. (c) Quantitative morphometric analysis of inflammatory cell infiltration based on H&E sections. (d) Quantitative assessment of collagen deposition and maturation within the wound bed derived from Masson's trichrome staining. (e) Representative H&E‐stained images of major organs. (*n* = 3, ^****^ *p* < 0.0001, ^***^ *p* < 0.001, ^**^ *p* < 0.01, ^*^ *p* < 0.05, ns: non‐statistical differences). Data are presented as mean ± SD. All images within the same image set were acquired at the same magnification and that the scale bar shown in the final image applies to the entire set.

Hematoxylin and eosin (H&E) staining (Figure 5a) reveals that the Ce‐BNC‐treated group maintains a largely intact and continuous epithelial architecture, exhibiting negligible signs of persistent infection. In stark contrast, the untreated infection groups display fragmented epithelial structures, as are characterized by dense inflammatory cell infiltration (hyperchromatic nuclei) and extensive liquefactive necrosis [59]. Quantitative analysis confirms that while the inflammatory cell density in the infection‐only group increased by approximately 50% compared to the healthy control (*p* < 0.0001). However, treatment with Ce‐BNC significantly attenuated this response, restoring inflammatory parameters to levels statistically comparable to the control group (Figure 5c). Furthermore, Masson's trichrome staining was utilized to assess the remodeling of the extracellular matrix (Figure 5b). In the Ce‐BNC group, the wound bed was characterized by a dense, organized network of newly formed collagen fibers (stained blue) beneath the regenerated epithelium. Quantitative morphometry indicates that the collagen‐positive area in the Ce‐BNC group reaches 28 ± 0.856% (Figure 5d), with fibers exhibiting a significantly more robust and uniform distribution compared to the sparse and disordered collagen arrangement in the other experimental groups. These findings suggest that Ce‐BNC effectively mitigates the local inflammatory microenvironment while significantly accelerating structural tissue regeneration.

Crucially, histological examination of the vital organs reveals no discernible structural abnormalities or systemic toxicity on the 10th day and 28th day post‐surgery (Figure 5e and Figure S20). In addition, study further quantified the residual Ce content in the heart, liver, spleen, lung, and kidney using ICP‐MS. Notably, the Ce levels in all of these organs at 28 days post‐administration are negligible, as shown in Table S5. These findings suggest that Ce‐BNC does not undergo significant long‐term retention or organ accumulation under the current experimental conditions and is likely cleared from the body over time.

### 2.5 Antibacterial Performance and Biofilm Eradication of Ce‐BNC SAzyme

#### 2.5.1 Broad‐Spectrum Antibacterial Activity and Biofilm Clearance

Following the confirmation of the pH‐switchable catalytic mechanisms of Ce‐BNC, study systematically evaluated its antibacterial potency in vitro. To ensure the high POD‐like activity of Ce‐BNC, Ce‐BNC was dissolved in phosphate‐buffered saline (PBS) at pH 3.8. Subsequently, H_2_O_2_ was added to simulate the inflammatory microenvironment, yielding a final concentration of 0.1 mmol L^−1^ (0.1 mm) [9]. *Staphylococcus aureus* (*S. aureus*), *Escherichia coli* (*E. coli*) and *Porphyromonas gingivalis* (*P. gingivalis*) were selected as representative *Gram‐positive*, *Gram‐negative* and *a major oral pathogen*, respectively. To differentiate the catalytic bactericidal effect from the inherent toxicity of H_2_O_2_, pristine H_2_O_2_ was employed as a control.

**FIGURE 6:**
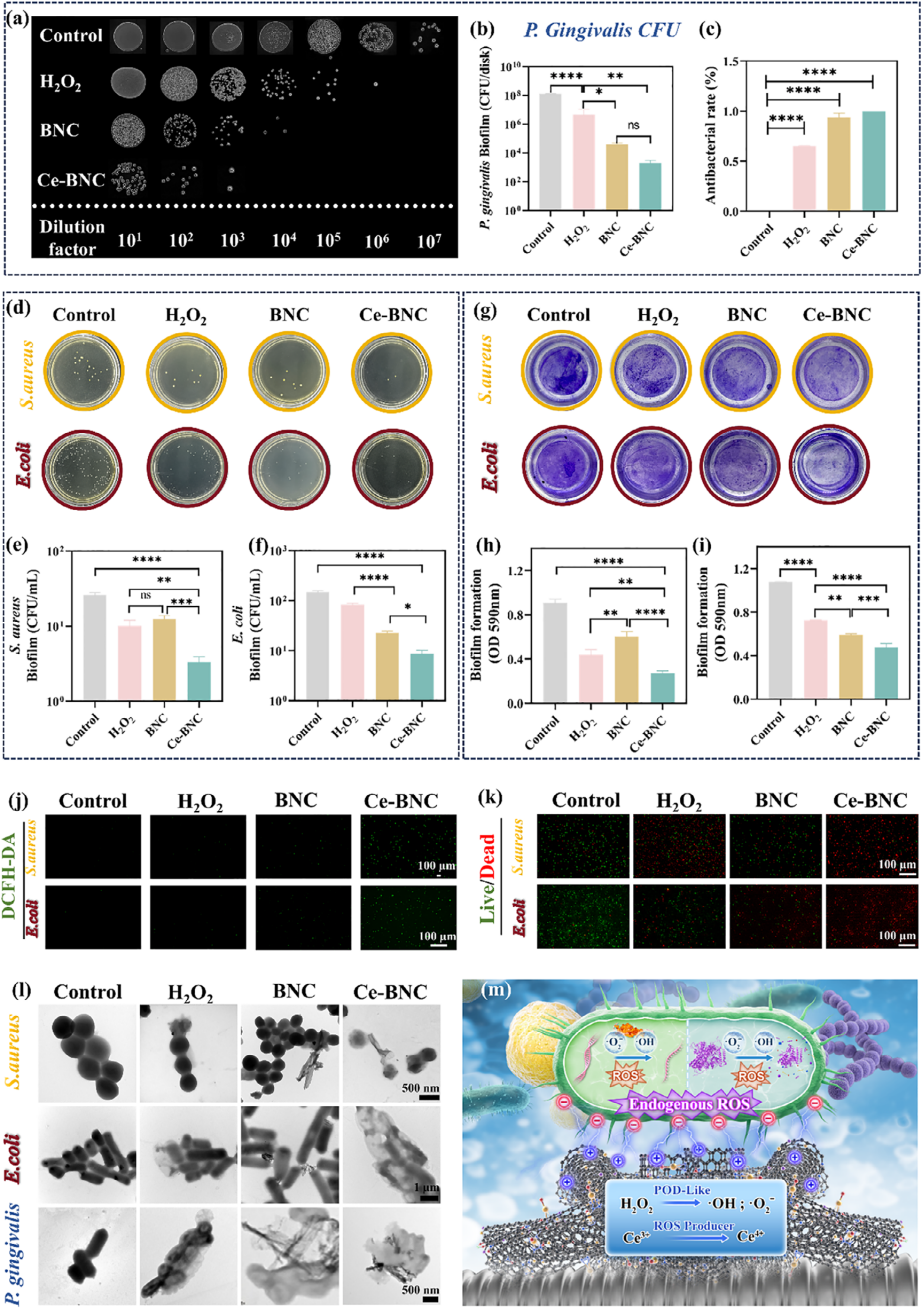
Antibacterial and anti‐biofilm performance of the Ce‐BNC SAzyme. (a,d) Representative pictures of *S. aureus*, *E. coli* and *P. gingivalis* colonies on agar plates following various treatments. Quantitative analysis (CFU counts) for (e) *S. aureus*, (f) *E. coli*, and (b) *P. gingivalis*. (c) Calculated antibacterial rate against the anaerobic pathogen *P. gingivalis*. (g) Visual representation of *S. aureus* and *E. coli* biofilm formation on agar plates across various treatment cohorts, and statistical analysis of (h) *S. aureus* and (i) *E. coli* biofilm colonies. (j) Intracellular ROS staining (DCFH‐DA staining) of *S. aureus* and *E. coli*. (k) Live/Dead staining images of *S. aureus* and *E. coli*. (l) TEM‐based morphological characterization of *pathogens* treated with distinct experimental groups. (m) Schematic diagram of antimicrobial mechanism of Ce‐BNC. The final working concentrations for H_2_O_2_ and Ce‐BNC are 0.1 mM and 70 µg mL^−1^, respectively. Statistical significance was determined via one‐way ANOVA (*n* = 3, ^****^ *p* < 0.0001, ^***^ *p* < 0.001, ^**^ *p* < 0.01, ^*^ *p* < 0.05, ns: non‐statistical differences).

The eradication of recalcitrant dental plaque biofilms remains a major challenge in PI therapy. Serial dilution plating shows that Ce‐BNC yielded the fewest viable *P. gingivalis* colonies among all groups (Figure 6a). CFU quantification reveals that Ce‐BNC reduces the bacterial burden from approximately 10^8^ to 10^3^ CFU per disk, corresponding to nearly a five‐order‐of‐magnitude decrease (Figure 6b). Although H_2_O_2_ and BNC alone exhibit antibacterial activity, both are less effective than Ce‐BNC. Consistently, Ce‐BNC achieves an antibacterial rate of nearly 99%, exceeding those of the H_2_O_2_ and BNC groups (Figure 6c). This enhanced performance is attributed to bimodal catalytic action: the POD‐like activity generates bactericidal ROS, while the CAT‐like activity produces O_2_, which effectively disrupts the anaerobic microenvironment essential for *P. gingivalis* survival. The broad‐spectrum antibiofilm activity of Ce‐BNC was further evaluated using *S. aureus* and *E. coli*. Colony images and Colony Forming‐Unit (CFU) quantification shows that Ce‐BNC markedly reduces the number of viable bacteria recovered from both biofilms compared with the control, H_2_O_2_, and BNC groups (Figure 6d–f). For *S. aureus*, H_2_O_2_ and BNC produce only moderate reductions in viable bacterial counts, whereas Ce‐BNC resulted in the lowest CFU level. A similar trend was observed for *E. coli*, for which Ce‐BNC significantly outperformed both H_2_O_2_ and BNC. As shown in Figure 6g‐i, while the BNC group exhibits some anti‐film activity, the Ce‐BNC SAzyme group demonstrates statistically significant superiority (*p* < 0.0001). This finding aligns with Ce‐BNC SAzyme's enhanced POD activity compared to BNC.

Because the initial experiments were performed at pH 3.8, corresponding to the optimal POD‐like activity of Ce‐BNC, CFU assays (Figure S21) were further conducted against all three bacterial strains at pH 6.5 to better approximate the mildly acidic PI infection microenvironment. In addition, to distinguish the catalytic antibacterial effect of Ce‐BNC from physical adsorption and nonspecific biological effects, an inactive Ce‐site counterpart named I‐Ce‐BNC and a supernatant control were introduced named S‐Ce‐BNC. The I‐Ce‐BNC shows modest antibacterial activity, which is higher than that of the untreated control but lower than that of BNC, whereas Ce‐BNC exhibits the strongest bacterial killing. This result suggests that the support itself may contribute a limited physical adsorption, but the enhanced antibacterial performance of Ce‐BNC primarily depends on the catalytically active Ce single‐atom sites. In addition, bacteria incubated with the supernatant collected after Ce‐BNC and H_2_O_2_ co‐incubation (S‐Ce‐BNC) exhibit substantially weaker inhibition than those directly treated with the Ce‐BNC/H_2_O_2_ system, indicating that the observed antibacterial effect could not be attributed mainly to physical adsorption or nonspecific soluble components, but instead required interfacial catalytic reactions.

**FIGURE 7:**
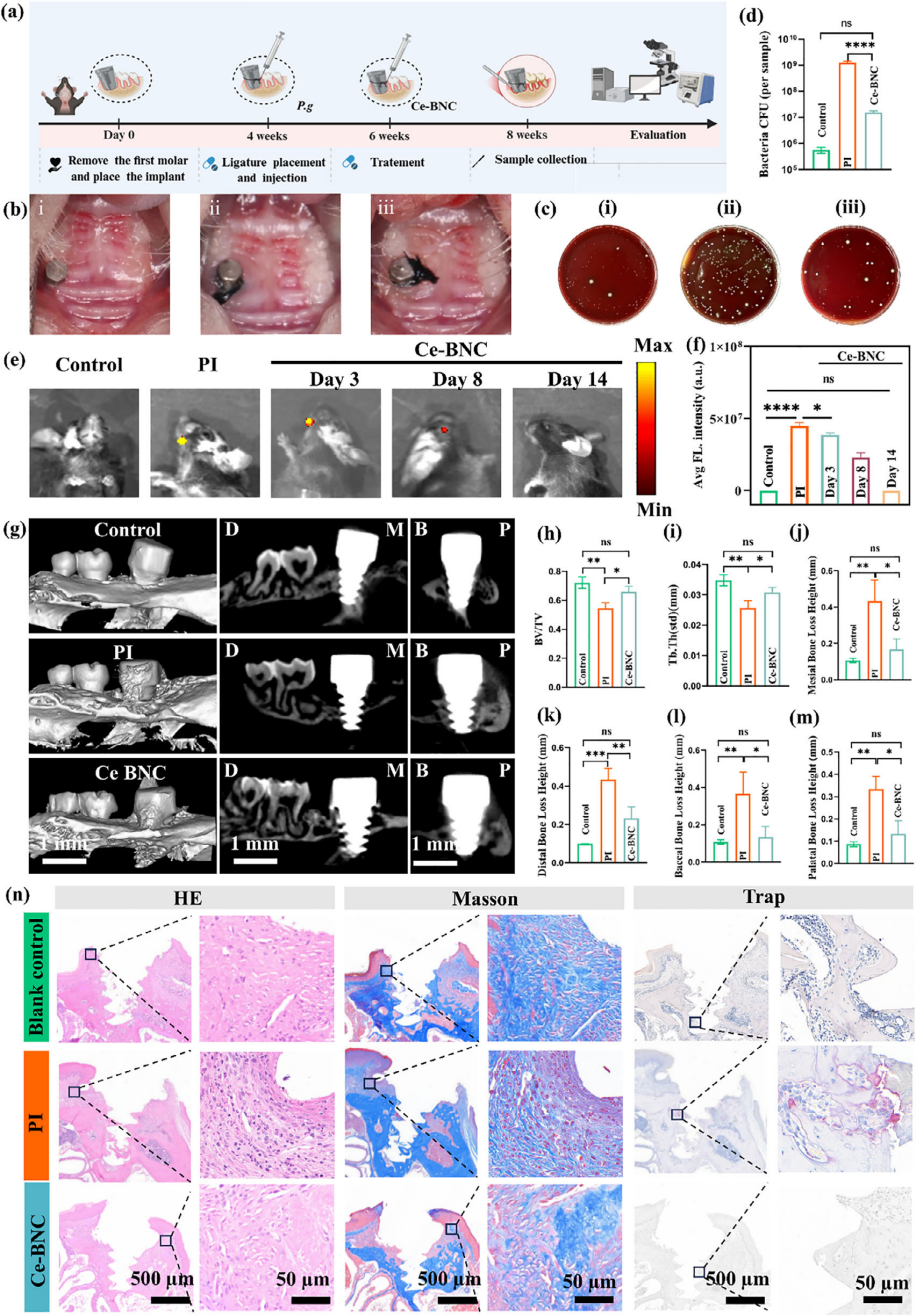
Therapeutic efficacy of the Ce‐BNC SAzyme platform within a murine PI microenvironment. (a) Methodological workflow for model construction and drug delivery. (b) Representative clinical phenotypes of mice on day 14 post‐treatment. (c) Photograph records of microbial outgrowth recovered from the peri‐implant sulcus and (d) corresponding quantitative analysis of CFUs. (e) In vivo fluorescence imaging of ROS level at the site of implants with different treatments on days 3, 8, and 14, and (f) the corresponding quantification. (g) Representative Micro‐CT 3D reconstructions and cross‐sectional images of the maxillae. (h‐m) Comprehensive morphometric analysis of key structural indices characterizing the alveolar bone. (n) Representative images of the H&E, Masson and TRAP staining images of PI models. Statistical analysis was performed via one‐way ANOVA. Data are shown as mean ± SD (*n* = 3) (^*^ *p* < 0.05, ^**^ *p* < 0.01, ^***^ *p* < 0.001, and ^****^ *p* < 0.0001). All images within the same image set were acquired at the same magnification and that the scale bar shown in the final image applies to the entire set.

Ce‐BNC retained pronounced antibacterial activity under pH 6.5, consistent with the POD‐like activity assay showing that approximately 80% of its catalytic activity is preserved at pH 6.5 (Figure 2c,d). Furthermore, CFU enumeration of microorganisms recovered from the peri‐implant sulcus confirmed the strong in vivo antibacterial effect of Ce‐BNC (Figure 7c,d). Collectively, these results support that the antibacterial activity of Ce‐BNC arises predominantly from physical adsorption and pH‐switchable Ce single‐atom catalysis and remains effective under physiologically relevant mildly acidic conditions.

#### 2.5.2 Mechanistic Insights Into Oxidative Damage and Membrane Disruption

To verify that the bactericidal effect was driven by POD‐mediated ROS generation, intracellular ROS levels were assessed using DCFH‐DA staining (Figure 6j). The Ce‐BNC group exhibits the most intense fluorescence, confirming the efficient conversion of exogenous H_2_O_2_ into intracellular oxidative stress. Live/dead staining (Figure 6k) corroborates these findings, showing a direct correlation between ROS production and bacterial mortality. To quantitatively evaluate the ROS‐generating capability of Ce‐BNC during antibacterial treatment, intracellular ROS levels in *S. aureus* and *E. coli* were analyzed by flow cytometry using DCFH‐DA fluorescent probe. As shown in Figure S22, the proportion of ROS‐positive *S. aureus* increased from 1.97% in the control group to 8.43% and 9.42% after treatment with H_2_O_2_ and BNC + H_2_O_2_, respectively. Notably, Ce‐BNC + H_2_O_2_ further increases the ROS‐positive population to 17.4%, which is significantly higher than that induced by H_2_O_2_ alone (^****^ *p* < 0.0001). By contrast, the inactive Ce‐site counterpart, I‐Ce‐BNC + H_2_O_2_, exhibits only 2.46% ROS‐positive cells. A similar trend was observed in *E. coli*. The proportions of ROS‐positive cells were 2.79%, 14.20%, 22.60%, 32.00%, and 4.27% in the control, H_2_O_2_, BNC + H_2_O_2_, Ce‐BNC + H_2_O_2_, and I‐Ce‐BNC + H_2_O_2_ groups, respectively. Although BNC + H_2_O_2_ increases intracellular ROS to some extent, Ce‐BNC + H_2_O_2_ produces the highest ROS level and was significantly more effective than H_2_O_2_ alone (^****^ *p* < 0.0001). In contrast, inactivation of the Ce catalytic sites almost completely abolished this ROS‐amplifying effect. These results support that the active Ce sites in Ce‐BNC promote H_2_O_2_‐dependent intracellular ROS generation in bacteria, thereby contributing to its catalytic antibacterial activity.

TEM shows that after treatment with the Ce‐BNC SAzyme, severe adhesion between cells and the SAzyme can be observed, accompanied by severely lysed of the bacterial cell membrane (Figure 6l). At the same time, Ce‐BNC shows a higher antibacterial effect against *E. coli* than against *S. aureus*. Such observations stem from the intrinsic architecture of *S. aureus*, which possesses a significantly thicker and more rigid peptidoglycan cell wall [60]. Therefore, it is believed that the *Gram‐negative* bacterium *E. coli* is more sensitive to this physical damage [61]. This potent antibacterial action is driven by robust electrostatic attraction between the positively charged Ce single‐atom sites and the negatively charged bacterial membranes. This interaction facilitates direct physical compromise of the cell envelope, an effect independent of any ROS‐mediated POD‐like catalytic activity. After the addition of H_2_O_2_, the bactericidal rate of Ce‐BNC reached 99.9%, which is attributed to the POD‐like activity that catalyzes H_2_O_2_. The ·OH and ·O_2_^−^ radicals generated by electron transfer chain reactions are closely associated with the insertion into the phospholipid bilayer, leading to conformational disorders in transmembrane proteins [62], accompanied by bacterial content leakage. More importantly, free radicals block bacterial nucleic acid metabolism.

Combine the above, study infer that the antibacterial mechanism of the Ce‐BNC SAzyme is as follows (Figure 6m): the Ce‐BNC SAzyme targets and adsorbs onto the surface of bacteria through electrostatic adsorption and subsequently catalyzes H_2_O_2_ to generate ·OH and·O_2_^−^. These mainly occur in the bacterial cell membrane and unsaturated phospholipid molecules. Phospholipid oxidation produces lipid peroxides that degrade the biofilm matrix. This disruption breaks down the structural barrier, allowing for heightened ionic flux across the membrane [63]. Beyond direct bactericidal effects, ROS effectively dismantles biofilms by oxidizing the extracellular polymeric substances (EPS) layer. This process involves the oxidative modification of nucleic acids and proteins, thereby neutralizing the protective barrier of the bacterial community [64]. Therefore, it can be seen that the inhibiting nucleic acid metabolism efficient bactericidal performance of the Ce‐BNC SAzyme is the result of the synergistic effect of physical contact damage and significant POD‐like activity in generating free radicals. For anaerobic bacteria, the O_2_ generated by Ce‐BNC's CAT‐like activity, which disrupts the anaerobic environment of *P. gingivalis* and achieving enhanced antibacterial efficacy.

### 2.6 Ce‐BNC SAzyme Targets the JAK‐STAT Signaling Axis via Inhibiting M1 Macrophage Polarization to Treat PI

#### 2.6.1 Antibacterial and Anti‐Inflammatory Performance of Ce‐BNC In Vivo

Following preliminary evaluations of Ce‐BNC antimicrobial and wound‐healing properties in skin experiments in vivo (Figure S23), study further established a C57BL/6 murine model of PI to systematically investigate the therapeutic potential of Ce‐BNC (Figure 7a). Detailed experimental procedures are available in the Supporting Information. Successful induction of PI was confirmed by typical inflammatory clinical manifestations, including pronounced gingival edema, dark‐red discoloration of the mucosa, localized food debris accumulation and bleeding upon probing (BOP). With a two‐week intervention, clinical photography documented the oral condition (Figure 7b). Image results show that the gingiva in the control group is in good condition, while the PI group presents obvious clinical manifestation, including gingival recession leading to clear exposure of implant threads, as well as significant redness and hyperemia of the gingival tissue. Although minor recession persisted, the color, morphology and texture of the gingival tissue in the Ce‐BNC‐treated mice are restored to levels comparable to the healthy control. Subgingival plaque analysis via CFU counting (Figure 7c,d) further confirms the potent microbial clearance capacity of Ce‐BNC. The pathogenic bacterial load in the Ce‐BNC group is significantly lower than that of the PI group (*p* < 0.001), with no statistical difference from the blank control, validating the efficacy of Ce‐BNC in eliminating peri‐implant biofilms in situ.

To investigate the dynamic changes of ROS in vivo, ROS fluorescence imaging was performed around the implanted sites at different time points after treatment. As shown in Figure 7e, the PI group exhibits the strongest fluorescence signal, indicating pronounced ROS accumulation and severe oxidative stress in the infected microenvironment. In the Ce‐BNC‐treated group, a detectable ROS signal was observed on Day 3, but its intensity was significantly lower than that of the PI group. The fluorescence intensity further decreased on Day 8 and was nearly diminished by Day 14. Quantitative analysis confirms a progressive reduction in ROS levels over time following Ce‐BNC treatment (Figure 7f). These findings demonstrate that Ce‐BNC effectively modulates excessive ROS in vivo, alleviates oxidative stress in the infected tissue microenvironment, and facilitates the resolution of inflammation and subsequent tissue repair.

To quantitatively evaluate alveolar bone preservation, three‐dimensional reconstruction and micro‐computed tomography (micro‐CT) analyses were performed on the peri‐implant maxilla (Figure 7g). As shown in Figure 7h, the PI group exhibits a marked reduction in bone volume fraction (BV/TV) compared with the control group, indicating substantial peri‐implant bone loss. Ce‐BNC treatment significantly increases BV/TV relative to the PI group, with no significant difference from the control group, suggesting effective preservation of alveolar bone volume. A similar trend was observed for trabecular thickness (Tb.Th) (Figure 7i). The PI group shows significantly thinner trabeculae, whereas Ce‐BNC treatment restored Tb.Th to a level close to that of the control group, demonstrating preservation of the trabecular microarchitecture.

Marginal bone loss was further quantified at the mesial, distal, buccal, and palatal aspects of the implant (Figure 7j–m). The PI group exhibits pronounced vertical bone loss at all measured sites, confirming severe multidirectional alveolar bone resorption. In contrast, Ce‐BNC treatment significantly reduces bone‐loss height in the mesial, distal, buccal, and palatal regions. Although slight residual bone loss remains, particularly at the distal site, no significant differences were observed between the Ce‐BNC and control groups. Collectively, these results demonstrate that Ce‐BNC effectively attenuates peri‐implant bone resorption while preserving both bone mass and trabecular integrity.

Histopathological evaluations provided further evidence of the dual role of Ce‐BNC in alleviating inflammation and promoting tissue regeneration (Figure 7n). H&E staining reveals characteristic areas of increased nuclear density in the PI group, indicating inflammatory activity. The Ce‐BNC intervention preserves the original cellular organization, exhibiting a morphological profile consistent with that observed in the blank control group. Quantitative immune response scoring confirms these observations (Figure S24), showing no significant difference in the degree of inflammation from baseline levels, with *p* < 0.001 compared to the PI group. Masson trichrome staining provides evidence of structural repair, with the control group exhibiting orderly and mature collagen deposition, while Ce‐BNC shows less collagen deposition than healthy peri‐implant tissues—likely due to that pathogen‐secreted collagenase caused the degradation of peri‐implant collagen tissue [65]. Notably, quantitative analysis reveals approximately a 1.5‐fold increase in collagen density compared to the PI group (*p* < 0.001) (Figure S24). Finally, tartrate‐resistant acid phosphatase (TRAP) staining quantified osteoclasts. In the PI group, the alveolar crest is irregular, showing numerous TRAP‐positive (TRAP+) osteoclasts and visible bone resorption pits. In contrast, the Ce‐BNC and control groups maintain smooth, continuous alveolar crests with minimal osteoclast presence (Figure 7n and Figure S24). The proportion of osteoclasts in the Ce‐BNC group (0.01 ± 0.06) is significantly lower than in the PI group (0.2 ± 0.16, *p* < 0.0001), confirming that the Ce‐BNC SAzyme suppresses osteoclast formation. Collectively, these results demonstrate that the profound anti‐inflammatory effect of Ce‐BNC is synergistically linked to microbial clearance and extracellular matrix repair, effectively resolving the complex pathology of PI.

#### 2.6.2 Transcriptomic Analysis of the Health and PI Mice Models

**FIGURE 8:**
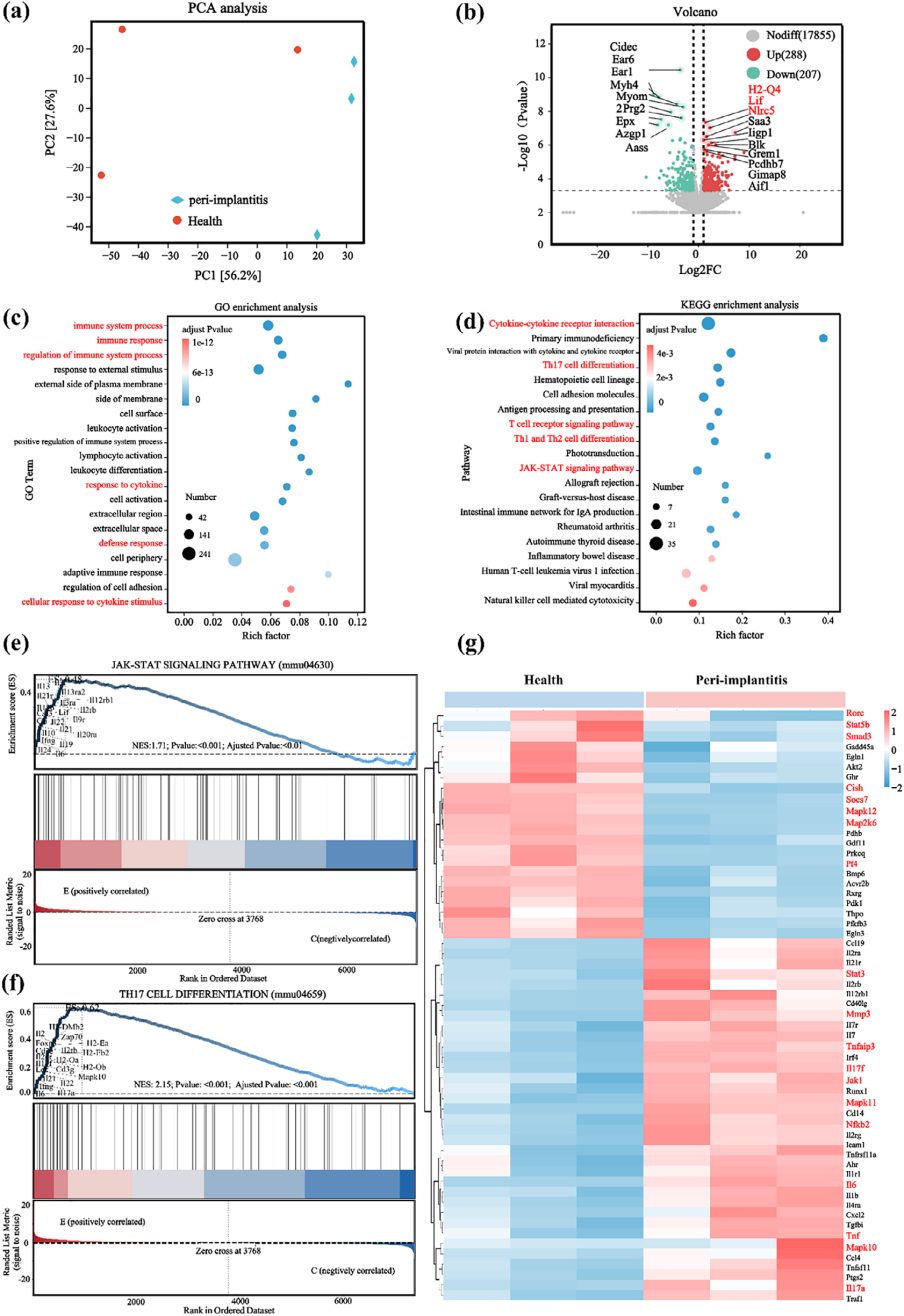
Transcriptomic analysis of the health and PI mice model. (a) PCA plot based on DEGs in gingival tissues. Discrete points represent individual samples from the healthy control and PI experimental groups. (b) Volcano plot displaying the differentially expressed genes (DEGs) between healthy and PI gingival tissues. (c,d) GO and KEGG enrichment analyses of the identified DEGs. (e) GSEA analysis revealed enrichment of gene sets associated with the JAK‐STAT pathway (f) and the Th17 pathway. (g) Heatmap of representative differentially expressed genes.

While the modulation of SAzyme macrophage polarization—specifically the inhibition of M1 phenotypes and the promotion of M2 states—is recognized as a hallmark of SAzyme‐mediated immune regulation, the underlying molecular mechanisms remain poorly understood. To elucidate the immune landscape of PI, we performed RNA sequencing (RNA‐seq) on gingival tissues from healthy and PI‐afflicted mice. Three independent biological replicates were included for each experimental group, with each replicate derived from an individual mouse. A marked distinction in the transcriptomic landscapes of healthy versus PI‐afflicted mice was observed via principal component analysis (PCA) (Figure 8a), suggesting fundamentally different gene expression modalities. Although a certain degree of variability was observed among the healthy samples, which likely reflects normal biological heterogeneity in gingival tissues, the PI and healthy groups remained clearly distinguishable in the PCA analysis, indicating distinct global transcriptional characteristics associated with disease. Subsequent screening yielded 495 DEGs, including 288 up‐regulated and 207 down‐regulated transcripts in the PI cohort (Figure 8b). Enrichment profiling (Gene Ontology (GO)/Kyoto Encyclopedia of Genes and Genomes (KEGG)) pinpoints adaptive immunity and inflammatory signaling as the predominant functional themes characterizing the top 20 altered pathways, specifically Th17 cell differentiation, the T cell receptor signaling pathway, Th1/Th2 cell differentiation, and the JAK‐STAT signaling pathway (Figure 8c,d).

Gene Set Enrichment Analysis (GSEA) results confirm a significant enrichment of both JAK‐STAT and Th17 signaling gene sets in PI‐exposed tissues (Figure 8e,f). The differentiation of Th1, Th2 and Th17 cells are critical for orchestrating inflammatory responses, as the activation of these pathways dictates macrophage polarization (M0 to M1/M2) and the subsequent balance between pro‐ and anti‐inflammatory cytokine secretion. Interestingly, while oxidative stress‐related pathways did not rank within the top 20, they exhibited significant modulation. Given that ROS act as secondary messengers in these cascades [66], their scavenging by Ce‐BNC likely serves as the primary trigger for the observed therapeutic reprogramming.

Detailed heatmapping (Figure 8g) further reveals that the PI group is characterized by the marked up‐regulation of key pro‐inflammatory mediators, including IL‐6, IL‐1β, IL1r1, TNF, IL‐17A, IL‐17F, and CXCL2. These factors are signature markers of M1‐type macrophage activation and Th17‐mediated inflammation [67]. Specifically, IL‐6 undergoes targeted binding with the surface receptors of RAW264.7 cells. This binding event induces a conformational change in the receptor, which subsequently recruits and activates the JAK family. This phosphorylation‐driven signaling axis correlates with the expression patterns of JAK1 and STAT3 (Figure 8g). The activation of the JAK‐STAT signaling pathway significantly upregulates gene expression of TNF, IL‐1β, and IL‐6 [68], which enhances inflammatory responses. Furthermore, alterations in genes associated with oxidative stress—such as Egln1, Gadd45a, Ahr, and Smad3—indicate a close relationship between ROS production and peri‐implant tissue damage during PI progression.

Based on these findings, we hypothesize that the pathogenesis of PI is driven by a feedback loop, in which P. gingivalis‐derived LPS triggers macrophage M1 polarization, mediating the de novo biosynthesis of the inflammatory signaling molecule IL‐6. This oxidative stress triggers an overactivation of JAK‐STAT signaling, thereby mediating the expression of downstream effectors, specifically pro‐inflammatory cytokines like TNF‐α and mediators of bone‐resorption, thereby perpetuating PI development and tissue loss. Consequently, the therapeutic efficacy of Ce‐BNC may stem from its ability to block this axis by restoring redox homeostasis, inhibiting M1 polarization, and normalizing JAK‐STAT signaling.

#### 2.6.3 Ce‐BNC SAzyme Targets JAK‐STAT Signaling Axis via Inhibiting M1 Macrophage Polarization to Treat PI

The pathogenesis of PI extends beyond simple bacterial invasion and represents a complex immune dysregulation process, in which pathogen‐induced oxidative stress triggers a persistent inflammatory cascade. Current nanozyme‐based strategies for PI treatment generally rely on either antibacterial or antioxidant functions, while lacking the ability to adapt to the heterogeneous and dynamically evolving peri‐implant microenvironment [69]. Herein, based on DFT calculations and systematic enzymatic characterization, the Ce‐BNC system was designed to achieve a pH‐switchable enzymatic relay by exploiting the intrinsic pH‐switchable catalytic behaviors of single‐atom Ce sites. Although the in vivo pH evolution during PI progression remains to be further quantitatively characterized, the acidic and relatively neutral microenvironments commonly associated with different pathological states provide a rational basis for designing pH‐switchable therapeutic systems. Therefore, Ce‐BNC provides an adaptive catalytic platform capable of responding to local pH variations and achieving stage‐dependent antibacterial and antioxidative regulation during PI treatment.

#### 2.6.4 Molecular Validation of Macrophage Phenotypic Reprogramming

**FIGURE 9:**
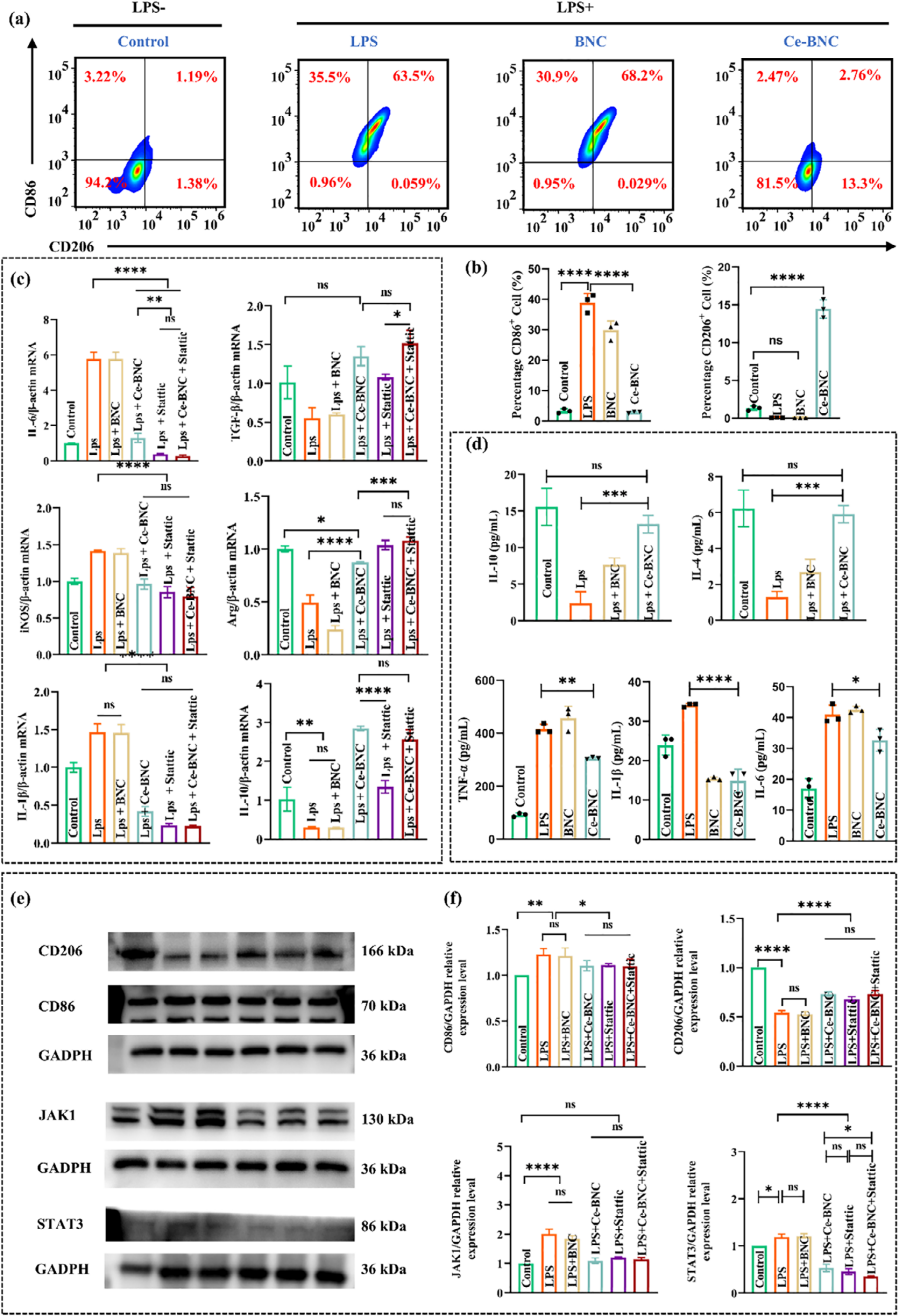
Ce‐BNC SAzyme modulates macrophage polarization and attenuates the JAK‐STAT signaling axis. (a) Cytometric analysis of macrophage polarization states and (b) numerical quantification of cells expressing M1‐specific (CD86) and M2‐specific (CD206) surface markers. (c) RT‐qPCR assessment of mRNA levels for M1‐phenotype and M2‐phenotype markers in treated macrophages. mRNA expression levels were normalized to the housekeeping gene β‐actin. Primer sequences are listed in Table S6. (d) The concentration of TNF‐α, IL‐1β, IL‐10, IL‐4 and IL‐6 in the supernatant after different treatments for 3 h. (e) Western blotting analysis and (f) corresponding densitometric quantification of key proteins associated with macrophage polarization and the JAK/STAT signaling pathway, with GAPDH as the internal loading control. Statistical analysis was performed using one‐way ANOVA. Data are shown as mean ± SD (*n* = 3) (^*^ *p* < 0.05, ^**^ *p* < 0.01, ^***^ *p* < 0.001, and ^****^ *p* < 0.0001).

To further validate the mechanism of Ce‐BNC in treating PI, study conducted polymerase chain reaction (PCR), enzyme‐linked immunosorbent assay (ELISA), western blotting, and flow cytometry to confirm the previously identified signaling pathways. Flow cytometry analysis was first performed to evaluate macrophage polarization (Figure 9a,b). LPS stimulation markedly promotes polarization toward the M1 phenotype, whereas Ce‐BNC treatment reduces the proportion of M1 macrophages and concomitantly increases the M2 population. Consistent with these findings, PCR analysis shows that LPS significantly upregulates the expression of the M1‐associated pro‐inflammatory mediator interleukin‐6 (IL‐6), interleukin‐1β (IL‐1β), and inducible nitric oxide synthase (iNOS) (Figure 9c). The increased production of these inflammatory factors may aggravate tissue damage and thereby accelerate PI progression. Although the BNC group also shows a moderate increase in the expression of these inflammatory mediators, their levels are substantially lower than in the LPS group. Notably, Ce‐BNC not only suppresses the expression of M1‐related inflammatory factors but also promoted, to some extent, the expression of the M2‐associated markers Arg‐1, TGF‐β, and IL‐10. The PCR results are therefore consistent with the macrophage polarization profiles obtained by flow cytometry.

The effects of Ce‐BNC on cytokine secretion were further evaluated by ELISA (Figure 9d). LPS stimulation substantially decreases the secretion of the anti‐inflammatory cytokines IL‐10 and IL‐4, whereas Ce‐BNC treatment restores their levels to values comparable to those of the control group. Conversely, LPS induces pronounced increases in the pro‐inflammatory cytokines TNF‐α, IL‐1β, and IL‐6. Ce‐BNC treatment significantly reduces the secretion of these cytokines compared with the LPS and/or BNC groups, although their levels are not completely restored to those of untreated control. Collectively, the flow cytometry, PCR, and ELISA results demonstrate that Ce‐BNC suppresses LPS‐induced M1 polarization while promoting an M2‐associated anti‐inflammatory phenotype. Moreover, these findings agree well with the SOD‐ and CAT‐like activity assays (Figure 2i,j) and the ROS‐scavenging assay (Figure 4d), further supporting the close association between excessive ROS accumulation and inflammatory activation. This demonstrates that Ce‐BNC effectively inhibits the production of pro‐inflammatory factors by neutralizing ROS‐induced inflammatory responses through SOD‐CAT‐like activity. Combined with Ce‐BNC's bacteriostatic effect, this characteristic enables preliminary therapeutic intervention targeting the pathogenesis of PI.

#### 2.6.5 Interception of IL‐6/JAK‐STAT Signaling Axis

RNA‐seq analysis identified the JAK–STAT pathway as a potential regulatory axis involved in the anti‐inflammatory effect of Ce‐BNC. Therefore, the anti‐inflammatory mechanism of Ce‐BNC was investigated by evaluating the expression levels of proteins associated with macrophage polarization markers CD86/CD206 and the JAK/STAT signaling pathway. Western blotting further showed that LPS increases CD86, JAK1, and STAT3 expression while reducing CD206, whereas Ce‐BNC reverses these changes (Figure 9e,f). Treatment with the STAT3 inhibitor Stattic produces a similar shift in the CD86/CD206 profile, and the combined treatment shows no clear additional effect, suggesting that Ce‐BNC and STAT3 inhibition may act through an overlapping pathway. PCR analysis shows that Ce‐BNC and the STAT3 inhibitor Stattic exerts similar regulatory effects on macrophage polarization. These results support the involvement of JAK1/STAT3 signaling in Ce‐BNC‐mediated macrophage polarization.

IL‐6 may link ROS regulation to JAK1/STAT3 signaling [70]. LPS markedly increases IL‐6 mRNA expression and secreted protein levels, whereas Ce‐BNC reduces both (Figure 9c,d). Given that excessive ROS can promote IL‐6 production and IL‐6 can activate the JAK1/STAT3 cascade, the ROS‐scavenging activity of Ce‐BNC may attenuate IL‐6‐driven signaling and thereby suppress M1‐associated inflammation. Thus, the present data suggest that Ce‐BNC regulates macrophage polarization, at least in part, through the ROS/IL‐6/JAK1/STAT3 axis.

**FIGURE 10:**
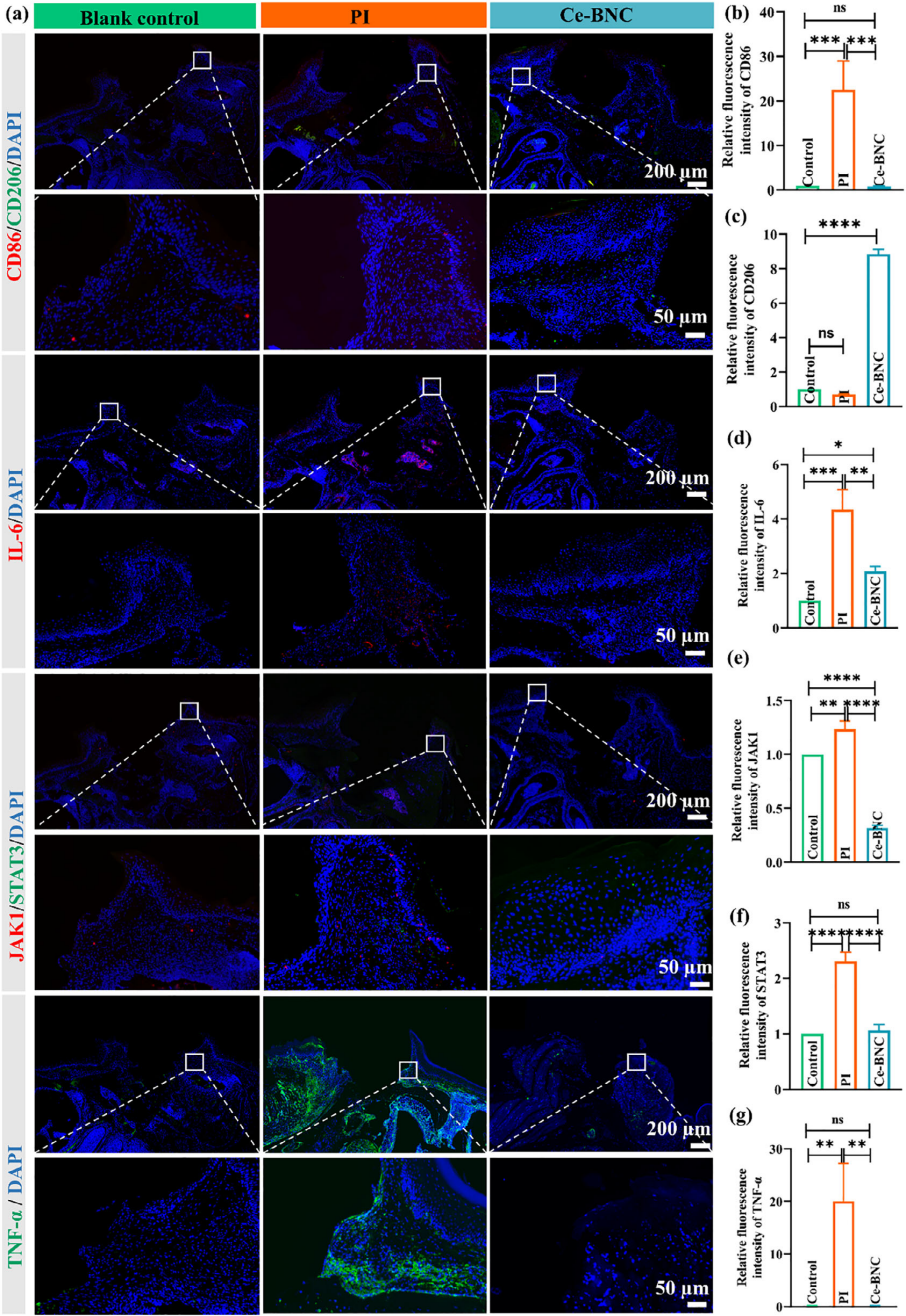
In situ IF evaluation of inflammatory markers and signaling transducers in peri‐implant tissues. (a) Representative IF images of CD86, CD206, IL‐6, JAK1, STAT3, TNF‐α and (b‐g) relative fluorescence intensities. Statistical significance was determined via one‐way ANOVA. Data is presented as Mean ± SD. (*n* = 3 independent biological replicates) ^*^ *p* < 0.05; ^**^ *p* < 0.01; ^***^ *p* < 0.001; ^****^ *p* < 0.0001; ns, no significance. All images within the same image set were acquired at the same magnification and that the scale bar shown in the final image applies to the entire set.

To further evaluate the effects of Ce‐BNC on oxidative stress and inflammatory pathways in peri‐implant tissues, we conducted immunofluorescence (IF) staining studies in vivo. The IF staining results visually demonstrate the intensity of CD86/CD206, IL‐6, TNF‐α and JAK1/STAT3 (Figure 10a). The high expression of CD86, IL‐6, TNF‐α, JAK1 and STAT3 in the PI group further validates the successful establishment of the inflammatory model and the previously proposed hypothesis of inflammatory pathways. Following local administration of Ce‐BNC, the proportion of CD206^+^ cells in the tissues significantly increased (*p* < 0.0001) (Figure 10c). The pronounced clusters of CD86, TNF‐α and IL‐6, which indicates a strong inflammatory response in the PI group, are notably diminished in the treated lesioned tissues (*p* < 0.01) (Figure 10b,d,g), indicating effective restoration of redox homeostasis. Meanwhile, quantitative analysis reveals that the expression levels of JAK1 and STAT3 are approximately 3‐fold and 1.5‐fold lower, respectively, compared to the PI group (Figure 10e,f).

Based on the experimental results and prior literature [71, 72], we propose the anti‐inflammatory mechanism of Ce‐BNC: when LPS binds to RAW264.7 cells, it induces M1 polarization. The resulting IL‐6 is released and activates JAK1 kinase, which phosphorylates STAT3. This STAT3 dimerization enters the nucleus, regulates target gene transcription, and subsequently directs M1 polarization, thereby initiating and amplifying the pro‐inflammatory response. In summary, macrophage polarization acts as a “regulator and effector” that bidirectionally modulates IL‐6 secretion and JAK/STAT activity. Polarized macrophages (M1/M2) not only serve as inflammatory effector cells but also regulate IL‐6 levels and JAK/STAT pathway activity through cytokine secretion, forming a closed‐loop regulatory mechanism. Our work shows that Ce‐BNC treatment reduces endogenous ROS production through SOD‐CAT‐like activities, effectively inhibiting the neutralizing closed‐loop regulation in the “M1 macrophage/IL‐6‐JAK/STAT” signaling pathway, thereby achieving a targeted strategy for treating PI.

#### 2.6.6 Conclusion

In summary, we developed a Ce‐BNC single‐atom nanozyme with intrinsic pH‐switchable enzymatic relay activity for adaptive PI therapy. Unlike conventional nanozymes with relatively fixed catalytic profiles, the atomically dispersed Ce sites in Ce‐BNC exhibit pH‐switchable catalytic behaviors arising from their local N/O coordination environment and reversible Ce^3+^/Ce^4+^ redox cycling. The coordination‐regulated electronic structure of Ce centers enables distinct substrate activation pathways under different pH conditions. This pH‐switchable catalytic selectivity enables Ce‐BNC to integrate antibacterial and anti‐inflammatory functions within a single nanozyme platform, thereby disrupting the vicious cycle of bacterial infection, oxidative stress, and immune dysregulation in PI. By alleviating excessive ROS accumulation, Ce‐BNC attenuates the M1 macrophage‐associated IL‐6/JAK/STAT inflammatory feedback loop, while CAT‐like oxygen generation contributes to hypoxia relief and further supports microenvironment remodeling and periodontal tissue protection. Furthermore, the stably coordinated Ce single atoms with low loading minimizes potential metal‐associated toxicity while maintaining efficient catalytic activity, providing a biosafe and adaptive nanozyme paradigm for inflammatory disease therapy. Although the pH‐ switchable catalytic switching of Ce‐BNC has been systematically validated, future studies involving in vivo microenvironment monitoring and pharmacokinetic evaluation will further elucidate its translational potential.

## 3 Experimental Methods

Experimental details are listed in the Supporting Information.

## Author Contributions

X.M.X., H.J.W., and L.N.L. executed the experiments and processed the data. Theoretical DFT calculations were designed and implemented by X.M.X. The initial manuscript was drafted by X.M.X., J.P.C., K.Q.S., and J.X.Y. The project's conceptualization and design were led by X.L., X.Y.L., and J.L. Supervision and final manuscript revisions were provided by X.L., X.Y.L., E.A., and Y.Y. All authors participated in result discussions and reviewed the final text.

## Funding

This work was supported by the Shandong Provincial Natural Science Foundation (ZR2021QH251; ZR2024MH308), the State Key Laboratory of Heavy Oil Processing (SKLHOP202403006) and with support from Key Laboratory of Applied Surface and Colloid Chemistry (Shaanxi Normal University), Ministry of Education (2024057).

## Conflicts of Interest

The authors declare no conflicts of interest.

## Supporting information




**Supporting File**: advs77002‐sup‐0001‐SuppMat.docx.

## Data Availability

The data that support the findings of this study are available from the corresponding author upon reasonable request.
