# Supplementary material for: A pH‐Switchable Cerium Single‐Atom Enzyme for Peri‐Implantitis Therapy via JAK‐STAT Signaling Axis‐Mediated Macrophage Reprogramming
Source: Adv Sci (Weinh). 2026 Aug 12;13(61):e77002. doi: 10.1002/advs.77002 (PMC13465578; doi:10.1002/advs.77002)
Supplement: Supplementary file 1 [file ADVS-13-e77002-s001.docx]

Supporting Information (SI)

**A pH-Switchable Cerium Single-Atom Enzyme for Peri-Implantitis Therapy via JAK-STAT Signaling Axis-Mediated Macrophage Reprogramming**

*Xiaomin Xia^1,2^*[*^†^*](https://pubs.rsc.org/en/content/articlelanding/2023/nh/d2nh00574c#fn1)*, Haojie Wu^3^*[*^†^*](https://pubs.rsc.org/en/content/articlelanding/2023/nh/d2nh00574c#fn1)*, Xingyun Li^3^**^*^, Lining Liu^3^, Kaiqing Song^1,2^, Jinpei Cui^1,2^, Jie Liu^1,2*^, Jiaxuan Yin^3^, Emtiaz Ahmed^4^, Yusuke Yamauchi^4,5*^, Xue Li^1,2*^*

1. **Materials and Methods**
   1.1. **Materials**

Carboxymethyl chitosan (CMCS; CAS: 83512-85-0; Mw 20 kDa; Mw: 150–800 kDa, Viscosity: 10–80 cst, the degree of carboxylation ≥ 80 %, the degree of deacetylation ≥ 85 %, purity ≥ 95 %) and tannic acid (TA; purity ≥ 95 %) were purchased from Beijing Solarbio Science & Technology Co., Ltd. (Beijing, China). Boric acid (purity ≥ 99 %), urea (purity ≥ 99 %), cerium nitrate hexahydrate (purity ≥ 99.5 %) was purchased from Shanghai Macklin Biochemical Co., Ltd. (Shanghai, China). Polyethylene glycol (purity ≥ 99.5 %, Sinopharm) were purchased from Sinopharm Chemical Reagent Co., Ltd (Beijing, China). Trypticase Soy Broth (TSB), Luria-Bertani broth (LB) and Brain-Heart Infusion Broth (BHI) were supplied by Beijing Solarbio Science & Technology Co., Ltd. (Beijing, China). The Live/Dead Cell Staining Kit was procured from Dalian Meilun Biotechnology Co., Ltd. (Dalian, China), while the Cell Counting Kit-8 (CCK-8) was sourced from Shanghai Macklin Biochemical Co., Ltd. (Shanghai, China).

**1.2. Material Preparation**

0.3 g of boric acid, 10.0 g of urea, 1.0 g of polyethylene glycol and 0.038 g of cerium nitrate hexahydrate as raw materials were added to the deionized (DI) water. The mixed solution was stirred at 120 ^o^C for 12 h to obtain a white powder, which was calcined at 900 ^o^C with a heating rate of 5 ^o^C/min for 6 h under Ar atmosphere to obtain Ce-BNC. BNC was prepared by the same method without the addition of cerium nitrate hexahydrate. CeO_2_-BNC with the same Ce loading as Ce-BNC were prepared by equal volume impregnating cerium nitrate hexahydrate into BNC, followed by calcination at 400 ^o^C with a heating rate of 5 ^o^C/min under air flow. To prepare I-Ce-BNC, Ce-BNC (20 mg) was immersed in 20 mL of 0.1 M NaH_2_PO_4_ solution and stirred at room temperature for 8 h to allow phosphate ions to coordinate with accessible Ce active sites. The resulting material was collected and thoroughly washed with deionized water by centrifugation at least five times to remove residual phosphate ions, yielding phosphate-blocked I-Ce-BNC^[1]^.

1.3. **Characterizations**

Nitrogen adsorption–desorption measurements were carried out on an Autosorb iQ3 analyzer. Prior to analysis, the samples were degassed at 200 °C for 10 h, and the isotherms were recorded at 77 K. The specific surface area was calculated using the Brunauer–Emmett–Teller (BET) method and the pore size distribution was derived from the Non‑Local Density Functional Theory (NL‑DFT) model. Powder X-ray diffraction (XRD) was performed with a Rigaku Ultima IV using Cu Kα (λ = 0.154178 nm) as radiation source. The 2*θ* range was set at 10-80^o^ with a counting rate of 5^o^ min^-1^. The field emission scanning electron microscopy (FESEM) was examined by JEOL JSM-7800F. The transmission electron microscopy (TEM) and selected-area electron diffraction (SAED) was recorded on JEOL JEM-2100 operated at 200 kV. Specifically, trace samples were dispersed in ethanol under ultrasonication and added evenly on the surface of silicon wafers, followed by drying with an infrared baking lamp. Aberration-corrected high-angle annular dark-field scanning transmission electron microscopy (AC-HAADF-STEM) images were obtained at 200 kV on JEOL JEM-ARM200F NEOARM equipped with aberration corrector for STEM (JEOL, Japan). X-ray photoelectron spectroscopy (XPS) was achieved on a PHI5000 Versaprobe III XPS spectrometer with an Al Kα X-ray source at the working conditions with basic vacuum degree of 6×10^-7^ kPa, working voltage of 10 kV and working current of 5 mA. Inductive Coupled Plasma (ICP) Emission Spectroscopy was carried out using an Agilent ICP-OES/730. Ce L_3_-edge extended X-ray absorption fine structure (EXAFS), and X-ray absorption near-edge structure (XANES) spectra were recorded in transmission mode. XANES and EXAFS data reduction and analysis were analyzed by Athena and Artemis software.

1.4. **Catalytic Performance Evaluation**

POD-like activity assays of BNC and Ce-BNC were performed using TMB as substrates. In brief, the material (BNC or Ce-BNC) was prepared into a 70 μg mL^−1^ solution using different pH (3.8, 5.5, 6.0, 6.5, 7.4) phosphate buffer solutions, and half of each was added to 1 mL ep tubes along with TMB at 37 ^o^C. After a certain reaction time, the absorbance of the color reactions was recorded after a certain reaction time using a UV-vis spectrophotometer. For catalytic stability testing, Ce-BNC was recovered after each reaction by washing, centrifugation, and drying, and reused for five cycles before reassessing its POD-like activity. After the final cycle, the recovered sample was collected and characterized by Aberration-Corrected TEM. The CAT-like activity was tested according to the principle that H_2_O_2_ was catalytically decomposed by BNC and Ce-BNC to generate O_2_ in PBS buffer (pH = 3.8, pH = 7.4 and pH = 10). The final concentrations of BNC and Ce-BNC and H_2_O_2_ in the system were 100 μg mL^−1^ and 10 mM. The concentration of dissolved O_2_ was measured with a dissolved oxygen meter (JPSJ-606L, Leici China). Electron paramagnetic resonance (EPR) was tested by FA-200 (JES) electron paramagnetic resonance spectrometer. According to the manufacturer’s instructions, the SOD-like activity detection kit was used to detect the SOD-like activity of BNC and Ce-BNC. Xanthine oxidase system was used to generate superoxide anion (·O_2_⁻), which reduced nitro blue tetrazolium (NBT) to formazan. ·O_2_⁻ could be removed through the SOD-like activity of BNC and Ce-BNC so that the formation of methyl was inhibited. The absorbance at OD560 was recorded. A Michaelis–Menten curve was produced by plotting the calculated initial rate against the substrate concentrations. The Michaelis–Menten equation was fitted to the data points to determine *K*_m_ and *V*_max_ by nonlinear least-squares regression analysis using Origin software. The above enzyme-like activity assays were independently repeated at least three times.

1.5. **Density Functional Theory (DFT) Calculations**

We used the DFT as implemented in the Vienna Ab initio simulation package (VASP) in all calculations. The exchange-correlation potential is described by using the generalized gradient approximation of Perdew-Burke-Ernzerhof (GGA-PBE). The projector augmented-wave (PAW) method is employed to treat interactions between ion cores and valence electrons. The plane-wave cutoff energy was fixed to 500 eV. Given structural models were relaxed until the Hellmann–Feynman forces smaller than -0.02 eV/Å and the change in energy smaller than 10^-6^ eV was attained. The vacuum thickness was set to be 20 Å to minimize interlayer interactions. During the relaxation, the Brillouin zone was represented by a Γ centered k-point grid of 7×7×1. Grimme’s DFT-D3 methodology was used to describe the dispersion interactions among all the atoms in adsorption models.

We constructed explicit solvation models to simulate the acidic and neutral reaction environments, in which solvent molecules and corresponding protonation states were introduced to represent the local reaction conditions. This approach was adopted because the local hydrogen-bonding network, solvent stabilization, and proton-transfer behavior under different pH conditions are difficult to accurately capture using empirical pH correction formulas alone.

The Gibbs free energy change (*ΔG*) was calculated by computational hydrogen electrode (CHE) model as follows:

$$\Delta G = \Delta E + \Delta ZPE - T \Delta S$$

where *ΔE* is the reaction energy obtained by the total energy difference between the reactant and product molecules absorbed on the catalyst surface and *ΔS* is the change in entropy for each reaction, *ΔZPE* is the zero-point energy correction to the Gibbs free energy.

The formula for calculating the formation energy as follows:

$$E_{\text{form}}=E_{\text{tot}}-E_{\text{rest}}-\sum_{i\in\text{1st coord.}} n_{i}E_{i}^{\text{iso}}$$

where: *E*_form_ represents the formation energy; *E*_ftot_ represents the total energy of the complete structure; *E*_rest_ represents the energy of the remaining structure excluding the first coordination environment; *E*^iso^_i_ represents the energy of the isolated atom of the i-th species in the first coordination environment; n_i_ represents the number of atoms of the i-th species.

1.6. **The Biological Safety and** **Antioxidant Properties**

*1.6.1 Cytotoxicity Test and Live/Dead Cell Staining*

To evaluate the cell compatibility of Ce-BNC, HGFs testing was performed using a cell counting kit (CCK-8). L929 and RAW264.7 cells were cultured in the well-prepared media for 24 h, 48 h and 72 h. After incubation in 37 ^o^C mediums for 24 h, live cells were determined by CCK-8 using a microplate reader at the OD_450_ (Bio-Tek, Winooski, VT, United States). Six wells were available in each experimental group. The morphology of HGFs was examined after culturing with different concentrations (50-100 μg mL^−1^) of Ce-BNC. Briefly, after co-culture washed three times with PBS, Subsequently, calcein-AM and PI were added to dye the live and dead cells for 30 mins. Cell morphology was examined using confocal laser scanning microscopy (CLSM, C2si, Nikon, Japan).

*1.6.2 Hemolysis Assay*

Fresh blood was collected from mice and added with anticoagulant and saline to test the hemolytic potential of nanoparticles. Blood diluted with distilled water was used as a positive control, and blood diluted with saline was used as negative control. The cells were incubated at 37 ^o^C for 4 h and centrifuged at 2,500 rpm for 10 mins, the supernatant was removed, and the absorbance at the OD_545_ was recorded using a microplate reader. Hemolysis rates were calculated according to the following formula:

$$\text{Hemolysis}\left（ \text{\%} \right）\text{=}\frac{\text{OD}_{\text{hydrogel}}\text{-}\text{OD}_{\text{negative}}}{\text{OD}_{\text{positive}}\text{-}\text{OD}_{\text{negative}}}\text{×100\%}$$

The assays were independently repeated at least three times.

*1.6.3 TEM Images of Ce-BNC Cellular Uptake*

Uptake and intracellular localization of Ce-BNC. After 24 h of treatment with Ce-BNC, RAW264.7 cells were washed three times with PBS, and cell precipitates were collected. These were fixed with 2.5% glutaraldehyde at 4°C for 24 h, washed three times with PBS, and then fixed with 1% osmic acid for 1 to 2 h. Finally, the samples were dehydrated with gradient concentrations of ethanol and propanol and were treated overnight with an embedded agent. The sections were then double-stained with lead citrate–uranyl acetate, and the slices were imaged using Hitachi HT-7800.

*1.6.4 Antioxidant Properties of BNC and Ce-BNC*

The ROS assay kit (Beyotime, Shanghai) was used to measure the ROS in cells treated with *lipopolysaccharide* (*LPS*) and nanoparticles. In short, RAW 264.7 cells were seeded into six-well plates at 3 × 10^4^ cells per well and divided into 5 groups: the first group was the blank control group. The second and third groups were treated with the positive control reagent for reactive oxygen species is Rosup and *P.g-LPS* (100ng mL^−1^) for 30 mins and 3 h to establish an *in vitro* inflammation model, respectively. Four to five groups were treated with *P.g-LPS* followed by the addition of safe concentrations (70 μg mL^−1^) of the BNC and Ce-BNC for 24 h. The medium containing nanoparticles was then replaced with serum-free medium supplemented with 10 μM DCFH-DA and incubated at 37 ^o^C for 30 mins. The cells were washed three times with serum-free medium and placed under an inverted fluorescence microscope for observation. The cells were then washed three times with PBS to remove the excess probe and immediately analyzed by flow cytometry using excitation at 488 nm and emission at approximately 525 nm. Intracellular ROS levels were quantified according to the percentage of the mean fluorescence intensity.

1.7. ***A*ntibacterial Properties**

*1.7.1 In vitro Antibacterial Properties*

*Escherichia coli* (ATCC 25922), *Staphylococcus aureus* (ATCC 25923), and *Porphyromonas gingivalis* (ATCC 33277) were used in this study. *E. coli* and *S. aureus* were cultured aerobically in LB/TSB at 37 °C for 18–24 h. *P. gingivalis* was cultured in pre-reduced supplemented BHI under strictly anaerobic conditions at 37 °C for 2–5 days. The anaerobic environment was maintained using an anaerobic chamber or anaerobic culture system.

*E. coli* and *S. aureus* colonies were selected from the fresh bacterial library mixed in pre-configured LB/TSB medium and grown under on a shaker at a speed of 200 rpm at 37 ^o^C for 24 h. Extract 1 mL from the above turbid solution and it was diluted with H_2_O_2_ (0.1 mmoL^-1^), BNC+ H_2_O_2_ (70 μg mL^−1^) and Ce-BNC+ H_2_O_2_ (70 μg mL^−1^) to an OD_630_ value of 0.06, and put into coculture for 24 h under the 80% N_2_, 10% H_2_ and 10% CO_2_. The solutions used to dilute the bacterial culture were prepared using solutions with pH 3.8 and 6.5, respectively. Additionally, I-Ce-BNC and S-Ce-BNC groups were included for experiments conducted at pH 6.5. For the S-Ce-BNC group, Ce-BNC was incubated with H_2_O_2_ at 37 °C for 30 min. After centrifugation, the resulting supernatant was collected and co-incubated with the bacteria. The bacterial solution after cocultivation with H_2_O_2_ (0.1 mmoL^-1^), BNC + H_2_O_2_ (70 μg mL^−1^) and Ce-BNC + H_2_O_2_ (70 μg mL^−1^) was diluted with PBS, and 20 μL of the above bacterial liquid was evenly spread on the agar plates and incubated in 37 ^o^C incubator for 24 h. *Porphyromonas gingivalis* (*P. gingivalis*) was cultured in anaerobic bags under the same experimental conditions as *S. aureus* and *E. coli*. Finally, the number of colonies in the petri dish was recorded and counted. Taking the co-cultivation of *E. coli* with BNC and Ce-BNC as an example, the method of co-cultivation of materials is the same as the previously described one. 0.5 mL of the bacterial suspension is aspirated and centrifuged at 6,000 rpm for 15 mins at room temperature. The obtained pellet was washed three times with sterile PBS and fixed with 2.5% glutaraldehyde for 2 h. The samples were observed by TEM. The bacterial biofilm experiment was cultured in the same way as before. The bacterial solution of different groups was placed in a 24-well plate with 2 mL per well for 24 h, and three double holes were set up in each group. The supernatant was gently aspirated with a syringe, the orifice plate was washed three times with PBS and then added to crystal violet solution and incubated at 37 ^o^C for 30 mins. The crystal violet solution was gently removed, and the biofilm ethanol dissolved. The OD value was measured by a microplate reader. The dried biofilm was photographed and recorded. The remaining bacteria after antibacterial treatment were resuspended using sterile saline and stained with a Live/Dead staining kit for 30 min, and the bacteria were observed by confocal microscopy. DCFH-DA staining was used to evaluate the ROS content of bacteria. Briefly, the treated bacteria were enriched by centrifugation, resuspended in saline with 10 µM DCFH-DA, and then incubated at 37 °C in the dark for 1 h, and ROS fluorescence was observed by confocal microscopy. The cells were then washed three times with PBS to remove the excess probe and immediately analyzed by flow cytometry using excitation at 488 nm and emission at approximately 525 nm. Intracellular ROS levels were quantified according to the percentage of the mean fluorescence intensity.

*1.7.2 In Vivo* Biocompatibility *and Antibacterial Performance*

The animal study was reviewed and approved by Institutional Animal Care and Use Committee of Qingdao University (approval number: No. *20240901BALB/c1520241220021*). Mice were kept in constant temperature (22 ^o^C), constant humidity (55%) and cyclic lighting (12 h light/12 h dark). For *in vivo* safety analysis and *in vivo* antibacterial experiment, twenty-four 6-8 weeks BALB/c male mice were purchased from Jinan Pengyue laboratory animal breeding *co. ltd*. A total of 24 mice were randomly allocated into four groups (n = 6 per group): Control, Infection, BNC, and Ce-BNC. Except for the Control group, mice in the other three groups were inoculated with *S. aureus* (10^6^ ~ 10^7^ CFU mL^-1^) to establish the cutaneous inflammatory infection model. After successful model establishment, mice in the Control, Infection, BNC, and Ce-BNC groups received subcutaneous injections of PBS, PBS, BNC, and Ce-BNC, respectively. The specific experimental steps are as follows: After anesthesia, a full-thickness wound with a diameter of 8 mm was made on the dorsal skin of the mice through aseptic operation. *S. aureus* solution was injected locally to make an infected wound model. After 24 h of infection, the wounds were treated in groups (PBS, BNC and Ce-BNC) every two days. The wound healing was observed, and the wound size was measured every two days. On the 10th and 28th day after infection, the mice were sacrificed and local skin tissue was obtained. Predefined exclusion criteria included unsuccessful model establishment, accidental death unrelated to the intervention, or severe complications requiring humane euthanasia. No animals were excluded from the final analysis. The skin tissues of each group were fixed with 10% neutral formalin solution for 24 h. The tissues were dehydrated and paraffin embedded. Tissue sections were made and HE and Masson were used for histological analysis. On the third day after treatment, sterilized cotton swabs were used to wipe the secretions from the skin wounds on the back of the mice. Soak the cotton swabs in PBS for 30 mins, then apply different groups of PBS to selective media plates. Place the medium in a bacterial incubator and incubate for 24 h before taking photos for recording. Histological examination of the vital organs was used to systemic evaluate toxicity on the 10th day and 28th day post-surgery. The residual Ce content in the heart, liver, spleen, lung, and kidney using ICP-MS.

1.8. **Establishment of Mouse** **Peri-implantitis Model**

*1.8.1. In Vivo Therapeutic Efficacy of Ce-BNC SAzyme In a Murine PI Model*

The study protocol of the mouse peri-implantitis model was approved by the Medical Ethical Committee of Stomatological Hospital affiliated with Qingdao University (NO*. 202412C5748202512194*). Briefly, 9 C57BL/6 mice (male, 6-8 w old, SPF) the right maxillary first molars of the mice were completely extracted, and implants were promptly inserted immediately after the extraction. Four weeks later, upon the formation of osseointegration of the implants, the mice were randomly allocated into 3 groups, namely Control, PI and Ce-BNC. With the exception of the control group, the remaining groups were subjected to treatment involving 5-0 silk thread ligation around the implant neck and inoculation of *P. gingivalis* for a period of two weeks to construct the PI model. Subsequently, the Control and PI groups remained untreated, whereas the Ce-BNC groups were administered with volume of 10 μL into the peri-implant lesion site every two days, Ce-BNC was administered *via* local in situ injection at a concentration of 70 μg/mL. *The ROS level of the peri-implant tissue was studied according to the similar methods in the literature*^[2]^*.* During two weeks of treatment, *the ROS probe DCFH-DA was intravenously injected at a dose of 1.8 mg/kg for 30 min. After that, the fluorescence imaging was recorded with an IVIS®Imaging System (excitation filter: 488 nm; emission filter: 525 nm, PerkinElmer).* After two weeks of treatment, the mice were euthanized, and the right maxillary bones were harvested for micro-CT scanning, pathological analysis and immunofluorescence staining. Using a dental probe, subgingival plaque from the implant was scraped and dispersed in pre-cooled PBS. The suspension was then subjected to gradient dilution followed by CFU counting.

When calculating the height of implant bone resorption, since the mesial and distal bone heights of the implant were flush with the crest of the alveolar ridge of the adjacent maxillary second molar in mice at the initial implantation of the implant,

The heights of bone recession at the mesial and distal ends of the implant were measured, respectively. 3D reconstruction and data analysis of MICRO CT: The original image was reconstructed using Recon 3D reconstruction software, and the target ROI was analyzed with Avatar data analysis software. All samples were analyzed in the same region to obtain required parameter values and export the data.

About long-term biosafety evaluation, following the establishment of PI, the animals received repeated treatment over a 28-day period. On day 28, the mice were sacrificed, and the heart, liver, spleen, lungs, and kidneys were collected. Major organs were subjected to hematoxylin and eosin (H&E) staining for histopathological evaluation, while the Ce contents were quantified by inductively coupled plasma mass spectrometry (ICP-MS).

*1.8.2. RNA Sequencing and Bioinformatics Analysis*

Frozen tissue samples were quickly immersed in liquid nitrogen and subsequently ground into a fine powder. Total RNA was extracted using TRIzol reagent, and its concentration was measured using a UV spectrophotometer. Digital gene expression (DGE) profiling was performed by Personalbio Technology Co., Ltd. (Shanghai, China). The concentration and purity of total RNA were determined using a NanoDrop spectrophotometer (Thermo Scientific NanoDrop one, Thermo Fisher Scientific, Waltham, MA, USA), while RNA integrity was assessed through either RNA-specific agarose gel electrophoresis or the Agilent Fragment Analyzer 5400 system (Agilent Fragment Analyzer 5400, Agilent Technologies Inc., California, USA).

mRNA enrichment was performed using the NEBNext® Poly(A) mRNA Magnetic Isolation Module (New England Biolabs Inc., Ipswich, Massachusetts, USA) with Oligo(dT) magnetic beads. The enriched mRNA was then randomly fragmented using divalent cations (ion interruption method) with the NEBNext Ultra II RNA Library Prep Kit for Illumina (New England Biolabs Inc., Ipswich, Massachusetts, USA). Using the fragmented mRNA as a template, double-stranded cDNA was synthesized with random oligonucleotides as primers (for strand-specific library construction, dUTP replaced dTTP in the dNTP mix during second-strand synthesis). The double-stranded cDNA was purified, followed by end repair, A-tailing, and adapter ligation. cDNA fragments of approximately 400–500 bp were size-selected using AMPure XP beads (Beckman Coulter, Beverly, CA, USA), PCR-amplified, and the PCR products were purified again with AMPure XP beads to obtain the final library. Library fragment sizes were analyzed using an electrophoresis-based instrument such as the LabChip GX. Total library concentration was measured using Pico green, and the effective concentration of the library was quantified by qPCR. Illumina Novaseq XPlus：The pooled libraries were serially diluted, quantified, and then sequenced on the Illumina NovaSeq X plus sequencing platform in PE150 mode.

Reference genomes and gene model annotation files are directly downloaded from the genomic website. The HISAT2 (v2.1.0) software is used to construct an index of the reference genome, and the paired-end clean reads are subsequently aligned to the reference genome using HISAT2. HISAT2 is chosen as the alignment tool because it can generate a spliced alignment database based on the gene model annotation files, thereby achieving better alignment performance compared to other non-splicing aligners.

Differentially expressed genes were then analyzed for KEGG pathway involvement and GO functional enrichment.

*1.8.3.* *Ce-BNC SAzyme Targets JAK-STAT Signaling Axis via Inhibiting Macrophage Polarization to Treat PI*

RAW 264.7 murine macrophages were cultured in Dulbecco’s Modified Eagle Medium (DMEM) supplemented with 10% fetal bovine serum (FBS) and at 37°C in a humidified atmosphere with 5% CO_2_. To induce an inflammatory M1 phenotype, cells were challenged with *lipopolysaccharide* (*LPS*, 100ng mL^-1^ for 12 h. For therapeutic evaluation, cells were treated with BNC or Ce-BNC SAzyme (70μg mL^-1^ in the presence of LPS.

*Quantitative Real-Time PCR (RT-qPCR)*

Total RNA was extracted using TRIzol reagent according to the manufacturer’s protocol. Complementary DNA (cDNA) was synthesized using a PrimeScript RT Master Mix. Quantitative PCR was performed using SYBR Green Master Mix on a Real-Time PCR System. The relative mRNA expression levels of M1 markers (IL-6, IL-1β, and INOS) and M2 markers (Arg-1, TGF-β, and IL-10) were calculated using the *2^−ΔΔCt^* method, with β-actin serving as the internal housekeeping control. Primer sequences are provided in table S6.

*Flow Cytometry Analysis*

To evaluate macrophage polarization, RAW 264.7 cells were harvested and washed with PBS. Cells were then stained with fluorophore-conjugated antibodies against CD86 (M1 marker) and CD206 (M2 marker) for 30 min at 4°C in the dark. After washing, the fluorescence intensity was measured using a flow cytometer. Data was analyzed to determine the percentage of CD86^+^ and CD206^+^ populations and the M2/M1 ratio.

*Enzyme-Linked Immunosorbent Assay (ELISA)*

The concentrations of pro-inflammatory cytokines, including tumor necrosis factor-alpha (TNF-α), interleukin-1 beta (IL-1β), interleukin-6 (IL-6), interleukin-4 (IL-4) and interleukin-10 (IL-10) in the cell culture supernatants were quantified using commercial ELISA kits following the manufacturer’s instructions. Absorbance was measured at the OD_450_ using a microplate reader.

*Western Blotting*

Total cellular proteins were extracted using RIPA lysis buffer containing protease and phosphatase inhibitors. Protein concentrations were determined using a BCA protein assay kit. Equal amounts of protein (30 μg mL^-1^) were separated *via* SDS-PAGE and transferred onto PVDF membranes. The membranes were blocked with 5% non-fat milk and incubated overnight at 4°C with primary antibodies against CD86, CD206, JAK1, and STAT3. After incubation with HRP-conjugated secondary antibodies, protein bands were visualized using enhanced chemiluminescence (ECL) reagent and quantified by densitometric analysis using ImageJ software, with GAPDH as the loading control.

*Statistical Analysis*

Statistical analysis was performed using one-way ANOVA. Data are shown as mean ± SD (n = 3) (**p* < 0.05, ***p* < 0.01, ****p* < 0.001, and *****p* < 0.0001).

**2. Discussion**

**2.1 Antibacterial Performance and Anti-infection Performance of Ce-BNC *In vivo***

According to previous literature, the surface of healthy and intact skin is mildly acidic, typically within a pH range of 4–6. Once the epidermal barrier is disrupted, the pH of an open wound may shift toward neutral or weakly alkaline values because of exposure to interstitial fluid and wound exudate.^[3,4]^ *In vitro* experiments have confirmed that in an acidic environment, Ce-BNC catalyzes the decomposition of H_2_O_2_ to generate ROS, showing good antibacterial performance. At a pH of 7.4, Ce-BNC catalyzes the decomposition of ·O_2_⁻ and H_2_O_2_ to produce oxygen and water, demonstrating good antioxidant effects. These findings inspire us to further explore the therapeutic effects of Ce-BNC on infected skin wounds *in vivo*.

For the *in vivo* bactericidal experiments, we established a mouse skin wound infection model, as shown in **Figure S23a**. On the second day, exudate around the wound of mice was taken to make bacterial blotting plate, and the results are shown in **Figure S23b**. The Ce-BNC group had the least colony attachment, and there is a statistical difference with the control group (**Figure S23c**). After grouped treatments, all groups show a trend of scab formation and contraction, except for the infection group on day 2. The increase in wound area after skin infection and wound inflammation may be related to a variety of factors, including the type of infection, the characteristics of the pathogen, the host's immune response and improper wound treatment, etc., which is consistent with the previous report by Xia et al.^[5,6]^ Overall, the Ce-BNC group has the fastest wound healing rate and shows a statistically significant difference compared to the control group (*p* < 0.0001) (**Figure S23d**, **S23e** and **S23f**). At the same time, the wound healing rate of the BNC group does not show better results than that of the control group, which may be due to BNC's inferior ability to scavenge ·OH and other ROS, leading to negative effects on normal tissues, like the cell compatibility shown in the Figure S16. The above results show that Ce-BNC system has good *in vivo* antibacterial activity and effectively reduces the inflammatory response caused by wound infection.

However, wound pH is highly dynamic and site-specific, varying with the anatomical location, wound type, inflammatory status, microbial composition, and healing stage. Therefore, the pH characteristics of cutaneous wounds cannot be directly generalized to infection sites in other tissues. For example, in peri-implantitis, bacterial metabolism within implant-associated biofilms can generate localized acidic microdomains, even when the surrounding tissue environment is near neutral.

**Supplementary Figure 1.** SEM image of BNC.

**Supplementary Figure 2.** N_2_ adsorption-desorption isotherms of Ce-BNC and BNC.

**Supplementary Figure 3.** The corresponding NL-DFT pore size distribution curves of Ce-BNC and BNC.

**Supplementary Figure 4.** TEM image of Ce-BNC with the corresponding selected-area electron diffraction (SAED) pattern shown in the inset.

**Supplementary Figure 5.** XRD patterns of Ce-BNC SAzyme and BNC.

**Supplementary Figure 6.** FTIR of Ce-BNC SAzyme and BNC.

**Supplementary Figure 7.** Aberration-corrected HAADF-STEM image of Ce-BNC SAzyme.

**Supplementary Figure 8.** DFT-optimized atomic structures and corresponding formation energies (*E_form_*) of Ce single-atom active sites with different O/N/B coordination environments on the BNC support.

**Supplementary Figure 9.** B 1s XPS spectra of Ce-BNC SAzyme.

**Supplementary Figure 10.** Differential charge density plot of CeN4O1. Yellow represents electron accumulation, whereas cyan indicates electron depletion, demonstrating that Ce serves as an electron-rich active site. Yellow spheres represent Ce atoms, pink spheres represent B atoms, gray spheres represent C atoms, blue spheres represent N atoms, and red spheres represent O atoms.

**Supplementary Figure 11.** (a) 3D stereoscopic view, (b) top view and (C) side view structures of Ce-BNC. Color codes: white: nitrogen; red: oxygen; yellow: cerium; brown: carbon; green: boron.

**Supplementary Figure 12.** (a) POD-like activity. (b) Ce-BNC SAzyme and BNC show POD-like activity by catalysing the oxidation of peroxidase substrates (TMB) to produce colorimetric reactions. (c) SOD-like activity. n = 3 independent experiments and data are presented as mean ± SD. (d) Time dependent oxygen generation in CAT-like activity.

**Supplementary Figure 13.** POD-like activity retention after five reaction cycles.

**Supplementary Figure 14.** The TEM and HAADF-STEM images of Ce-BNC SAzyme after five reaction cycles.

**Supplementary Figure 15.** Projected density of states (PDOS) of Ce and O atoms for the adsorbed intermediates involved in (a, d) POD-, (b, e) SOD-, and (c, f) CAT-like catalytic reactions under acidic and neutral conditions. (a-c) panels represent the PDOS under acidic and (d-f) represent the PDOS under neutral conditions. The dashed line indicates the Fermi level (0 eV).

**Supplementary Figure 16.** Vigor of L929 incubated with BNC (0, 50, 60, 70, 80, 90 and 100 μg mL^−1^) for 24 h, 48 h, and 72 h, respectively. A 96-well plate without BNC (0 μg mL^−1^) was used as a blank control. The solid line represents whether the experimental group is statistically different compared to the blank control. (n = 6, **** *p* < 0.0001, *** *p* < 0.001, ** *p* < 0.01, * *p* < 0.1, ns: non-statistical differences).

**Supplementary Figure 17.** (a: i–iii) Cell viability of RAW264.7 following incubation with Ce-BNC at varying concentrations (0, 50, 60, 70, 80, 90, and 100 μg mL^-1^) for 24, 48 and 72 h, respectively. Cells without Ce-BNC (0 μg mL^-1^) served as the blank control. Horizontal bars indicate statistical significance compared to the blank control. (n = 6, **p* < 0.05, ** *p* < 0.01, *** *p* < 0.001, **** *p* < 0.0001, ns: not significant). (b) Representative live/dead fluorescence staining images of RAW264.7 after treatment with Ce-BNC.

**Supplementary Figure 18.** Blood compatibility of Ce-BNC SAzyme.

**Supplementary Figure 19.** Flow cytometric analysis of intracellular ROS generation in RAW264.7.

**Supplementary Figure 20*.*** Representative H&E-stained images of major organs after administration 28 days. All images within the same image set were acquired at the same magnification and that the scale bar shown in the final image applies to the entire set.

**Supplementary Figure 21*.*** Representative pictures of (a) *P. gingivalis,* (b) *S. aureus* and (c) *E. coli* colonies on agar plates following various treatments. Quantitative analysis (CFU counts) for (d) *P. gingivalis*, (e) *S. aureus*, and (f) *E. coli*.

**Supplementary Figure 22. Flow cytometric analysis of intracellular ROS generation in bacteria.** Representative flow cytometry histograms and quantitative analysis of DCFH-DA-positive cells in (a, b) *S. aureus* and (c, d) *E. coli* after treatment with H_2_O_2_, BNC + H_2_O_2_, Ce-BNC + H_2_O_2_, or I-Ce-BNC + H_2_O_2_. Data are presented as mean ± SD. *****p* < 0.0001; ns, not significant.

**Supplementary Figure 23*.*** Antibacterial properties of Ce-BNC SAzyme *in vivo*. (a) Schematic diagram of the inflammation model on the dorsal skin of mice and the treatment process of Ce-BNC. (b) The colony formation images of *S. aureus* at different dilution factors. (c) CFU counts of *S. aureus* biofilms with different treatments. (d) Representative photographs of skin wounds at various stages of treatment with different treatments. (e) Schematic diagram of wound healing progression at different times. (f) Wound healing rates under different treatment groups. Wound healing rates in the different treatment groups on (g) day 2 and (h) day 8. Data were analyzed using one-way ANOVA. Data are presented as the mean ± SD (n = 6 mice per group). **p* < 0.05, ***p* < 0.01, ****p* < 0.001, and *****p* < 0.0001; ns, not significant.

**Supplementary Figure 24*.*** (a) Statistics of the number of immune cells. (b) Percentage of collagen volume fraction. (c) Statistics of the number of osteoclast cells.

**Supplementary table 1.** Porosity parameters of Ce BNC and BNC gained from nitrogen adsorption isotherms.

|  | SBET (m^2^/g) | Total Pore  Volume (cc/g) |
| --- | --- | --- |
| Ce BNC | 545.92 | 1.08 |
| BNC | 679.65 | 1.11 |

**Supplementary Table 2.** ICP-OES result for actual Ce content in Ce-BNC

| Catalyst | Ce content |
| --- | --- |
| Ce-BNC | 4 *wt*.% |

**Supplementary Table 3.** Elemental quantification of Ce-BNC determined by XPS spectra.

|  | B (*wt*.%) | N (*wt*.%) | C (*wt*.%) | O (*wt*.%) | Ce (*wt*.%) | Ce^3+^/Ce^4+^ |
| --- | --- | --- | --- | --- | --- | --- |
| Ce-BNC | 10.7 | 16.2 | 55.3 | 15.6 | 2.2 | 3.3 |

**Supplementary Table 4.** EXAFS fitting parameters at the Ce *L*_3_-edge for Ce-BNC. (S_0_^2^=0.94)

| Sample | Shell | CN^a^ | *R*(Å)^b^ | σ^2^(Å^2^)^c^ | Δ*E* (eV)^d^ | *R*-factor |
| --- | --- | --- | --- | --- | --- | --- |
| Ce-BNC | Ce-O | 1.37 | 2.46 ± 0.17 | 0.0115 ± 0.0132 | 4.5 ± 2.3 | 0.0117 |
|  | Ce-N | 3.90 | 2.61 ± 0.03 | 0.0115 ± 0.0132 |  |  |

CN^a^: coordination numbers; *R*^b^: bond distance; σ^2c^: Debye-Waller factors; Δ*E*^d^: the inner potential correction. *R* factor: goodness of fit.

**Supplementary table 5.** ICP-MS result for actual Ce content in organs.

| Organ | Heart | Liver | Spleen | Lung | Kidney |
| --- | --- | --- | --- | --- | --- |
| Ce contents /(μg g⁻¹) | 0.141 ± 0.005 | < LOD | 0.107 ± 0.006 | < LOD | < LOD |

Unit: μg g^-1^ (wet tissue weight); data are expressed as mean ± SD, n = 3; < LOD（below the limit of detection)

**Supplementary Table 6.** Primer sequences used in this study.

| Gene | Forward sequence (5’ to 3’) | Reverse sequence (5’ to 3’) |
| --- | --- | --- |
| β-actin | CATCCGTAAAGACCTCTATGCCAAC | ATGGAGCCACCGATCCACA |
| Arg | CTCCAAGCCAAAGTCCTTAGAG | AGGAGCTGTCATTAGGGACATC |
| IL-1β | TCCAGGATGAGGACATGA GCAC | GAACGTCACACACCAGCAGGTTA |
| IL-10 | ATGCTGCCTGCTCTTACTGACTG | CCCAAGTAACCCTTAAAGTCCTGC |
| IL-6 | CCACTTCACAAGTCGGAGGCTTA | CCAGTTTGGTAGCATCCATCATTTC |
| TGF-β | CTTCAGCCTCCACAGAGAA GAACT | TGTGTCCAGGCTCCAAAT ATAG |
| iNOS | CAGCTGGGCTGTACAAACCTT | CATTGGAAGTGAAGCGTTTC |

**References**

1. Y. Tang, X. Liu, P. Qi, et al., “Single-Atom Ce-Doped Metal Hydrides with High Phosphatase-like Activity Amplify Oxidative Stress-Induced Tumor Apoptosis,” *ACS Nano* 18 (2024): 25685–25694, https://doi.org/10.1021/acsnano.4c07851.

2. Z. Han, Y. Li, X. Zhan, et al., “A Versatile Nanoplatform with Excellent Biofilm Permeability and Spatiotemporal ROS Regulation for Peri-Implantitis Treatment,” *Theranostics* 15 (2025): 3490–3516, https://doi.org/10.7150/thno.108830.

3. Y. Meng, L. Chen, Y. Chen, et al., “Reactive Metal Boride Nanoparticles Trap Lipopolysaccharide and Peptidoglycan for Bacteria-Infected Wound Healing,” *Nature Communications* 13 (2022): 7353, https://doi.org/10.1038/s41467-022-35050-6.

4. N. Tang, Y. Zheng, X. Jiang, et al., “Wearable Sensors and Systems for Wound Healing-Related pH and Temperature Detection,” *Micromachines* 12 (2021): 430, https://doi.org/10.3390/mi12040430.

5. G. S. Schultz, R. G. Sibbald, V. Falanga, et al., “Wound Bed Preparation: A Systematic Approach to Wound Management,” *Wound Repair and Regeneration* 11 (2003): S1–S28, https://doi.org/10.1046/j.1524-475X.11.s2.1.x.

6. X. Xia, X. Song, Y. Li, et al., “Antibacterial and Anti-Inflammatory ZIF-8@Rutin Nanocomposite as an Efficient Agent for Accelerating Infected Wound Healing,” *Frontiers in Bioengineering and Biotechnology* 10 (2022): 1026743, https://doi.org/10.3389/fbioe.2022.1026743.
